# Morphological studies of rose prickles provide new insights

**DOI:** 10.1038/s41438-021-00689-7

**Published:** 2021-09-23

**Authors:** Ningning Zhou, Fabienne Simonneau, Tatiana Thouroude, Laurence Hibrand-Saint Oyant, Fabrice Foucher

**Affiliations:** 1 Univ Angers, Institut Agro, INRAE, IRHS, SFR QUASAV, F-49000 Angers, France; 2 National Engineering Research Center for Ornamental Horticulture; Flower Research Institute (FRI), Yunnan Academy of Agricultural Sciences, Kunming, 650231 China; 3 Univ Angers, INRAE, SFR QUASAV, F-49000 Angers, France

**Keywords:** Plant morphogenesis, Non-model organisms

## Abstract

Prickles are common structures in plants that play a key role in defense against herbivores. In the *Rosa* genus, prickles are widely present with great diversity in terms of form and density. For cut rose production, prickles represent an important issue, as they can damage the flower and injure workers. Our objectives were to precisely describe the types of prickles that exist in roses, their tissues of origin and their development. We performed a detailed histological analysis of prickle initiation and development in a rose F1 population. Based on the prickle investigation of 110 roses, we proposed the first categorization of prickles in the *Rosa* genus. They are mainly divided into two categories, nonglandular prickles (NGPs) and glandular prickles (GPs), and subcategories were defined based on the presence/absence of hairs and branches. We demonstrated that NGPs and GPs both originate from multiple cells of the ground meristem beneath the protoderm. For GPs, the gland cells originate from the protoderm of the GP at the early developmental stage. Our findings clearly demonstrate that prickles are not modified trichomes (which originate from the protoderm). These conclusions are different from the current mainstream hypothesis. These results provide a foundation for further studies on prickle initiation and development in plants.

## Introduction

Superficial tissues (epidermis) and appendage structures (trichomes, spinescences) of plant organs are the first lines of defense against multiple abiotic and biotic stresses. The basic terminologies of these appendages are frequently inaccurately cited in scientific reports, leading to confusion and difficulties in distinguishing the different terms. Some authors have described emergences as prickles, e.g., prickles on the stems or leaves of plants such as *Solatium torvium*, *Aiphanes acanthophylla*, and roses^1^, and some have referred to trichomes as emergences, e.g., grape emergences^2^. Another common source of confusion originates among prickles, thorns, and spines. Many plants described with thorns or spines actually have prickles^3–8^.

Depending on the presence of vascular bundles, we can divide these structures into two categories: (1) trichomes (Supplementary Fig. 1a and b) and prickles (Supplementary Fig. 1c and d), which are not vascularized and are generally easy to remove^9,10^; and (2) thorns (Supplementary Fig. 1e and f) and spines (Supplementary Fig. 1g), which have vascular bundles and cannot be easily separated from organs that have vascular tissues (spines, usually modified from leaves, and thorns, modified from stems or shoots) (Supplementary Fig. 1h)^11,12^. Thus, prickles can be easily distinguished from thorns and spines: mature prickles are outgrowths connected to the bark^13^, while thorns and spines are outgrowths connected to the phloem and the xylem^11,14^.

Confusion of trichomes and prickles is also common. Trichomes are epidermal appendages that originate only from the protoderm, and they are diverse according to their final forms and structures, locations, and functions^10,15^. They are mainly divided into nonglandular trichomes (NGTs) and glandular trichomes (GTs)^10^. Both types can be unicellular or multicellular and branched or unbranched. Presently, the genetic and molecular mechanisms of NGTs are well understood in *Arabidopsis thaliana*, and numerous related genes have been identified (reviewed by Hülskamp^16^ and Zhou^17^). These genes encode proteins belonging to the MYB, bHLH, WD40, WRKY, and C2H2 zinc finger protein families. A trimeric activator complex consisting of MYB (GLABRA1)-bHLH (GLABROUS3/ENHANCER OF GL3)-WDR (TRANSPARENT TESTA GL1) plays a key role in NGT initiation^16^. The genetic pathway for GT initiation is not yet well known (reviewed by Huchelmann et al.^18^ and Chalvin et al.^19^). In *Solanum*, an HD-ZIP IV transcription factor (WOOLLY) may interact with the B-type cyclin CycB2 and the C2H2 zinc-finger protein (HAIR) to induce GT initiation (reviewed by Chalvin et al.^19^). In *Artemisia annua*, an HD-ZIP IV transcription factor (AaHD8) may interact with a MIXTA-like protein (AaMIXTA1), which activates *AaHD1*, leading to GT initiation^20^.

Prickles are common structures in plants, which are involved in defense against insects and large mammalian herbivores^21^. The morphogenetic and molecular mechanisms underlying prickle initiation and development remain largely unknown. A few reports have described the anatomical structures of prickles^13^, especially in roses^9,22,23^. As the analyses were performed in late developmental stages, conclusions about the tissues from which prickles originate are difficult to draw, leading to different and controversial hypotheses developed below.

The mainstream hypothesis is that prickles originate from multiple cellular divisions of the epidermis^3,24,25^ and are considered as modified GTs, with lignification leading to a hard and sharp appendage^2,5,22,26^. Nonglandular prickles (NGPs) were described as a late stage of glandular prickles (GPs)^22^. Based on this hypothesis, molecular approaches were developed to test the trichome origin of prickles in rose and *Rubus*. A comparison of transcript accumulation between rose F1 genotypes with no, low-density (NGPs) and high-density (GPs and NGPs) prickles revealed significant differences for some candidate genes, such as *RcTTG2*^27^. Unfortunately, prickle types, NGPs and GPs, were mixed in the previous study. Later, Zhou et al.^28^ proposed that prickle and trichome initiation involve different genetic pathways, as no major difference was observed during prickle initiation for candidate gene homologs of genes known to control trichome initiation in *A. thaliana*. However, on the basis of a transcriptomic approach, the molecular network controlling prickle initiation was proposed to be similar in *Rubus*^29^ and in roses^30,31^ to the one described for trichome initiation in *A. thaliana*. The relationship between prickles and trichomes at the molecular level is still a source of debate. A precise histological description of the tissues is requested for clear conclusions.

Another hypothesis is that rose prickles are spines. Prickles were proposed to be modified leaves without internal vascular tissues, as the abscission cell layer of prickles resembled the abscission layer of deciduous leaves, with mature prickles easily peeled off^9^. However, no strong evidence was presented to support this hypothesis. Li et al.^23^ suggested that cells from the prickle abscission region were different from cells of the petiole abscission zone according to the anatomical structure and chemical composition of tender prickles.

Later, Angyalossy et al.^13^ defined prickles as “sharp outgrowths from the bark, without vascular tissue”, based on longitudinal sections through the developed prickles of *Polyscias mollis*, *Piptadenia gonoacantha*, and *Oplopanax horridus*. However, the “bark” term is unprecise, as it refers to all tissues exterior to the vascular cambium, including tissues such as the periderm (composed of cork, cork cambium, and phelloderm), cortex (comprising ground tissues), phloem and epidermis^32,33^. In conclusion, the origin of prickles in plants is still controversial and requires further investigation.

Wild roses belong to the genus *Rosa* in the family *Rosaceae*. The genus *Rosa* is composed of ∼200 species and is widely distributed in cold temperate to subtropical regions^34^. Rose is a such beautiful flower with wonderful fragrant, have always been popular at different periods and in many civilizations since it plays a part in many religions and has come to symbolize romance. Today, rose is one of the most economically important ornamental plants in the world. Most roses have prickles on their stems. For cut rose production, removing prickles is an essential step before packaging. This process causes wounding on the stem, largely affects transportation tolerance and vase life, and reduces the ornamental value. Furthermore, prickles represent a risk of injury to workers. Therefore, rose cultivars with many prickles are generally not accepted for the production of cut roses, even if they have other outstanding ornamental traits. In rose, prickles are very diverse, showing different types, shapes, sizes, densities, and colors. Furthermore, genetic resources such as several high-density SNP-based genetic maps from rose F1 populations^27,35,36^ and GWAS collections^27,37,38^ are available. The recent production of two high-quality reference genome sequences^27,39^ allows genomic approaches. Therefore, rose is a good model plant with which to study the molecular and genetic bases of prickle initiation and development.

In this study, our main objectives were to characterize in detail the initiation and development of prickles in roses using histological approaches and to investigate their diversity in terms of form. The major questions are as follows: (i) which types of prickles exist in roses? (ii) Which tissues do prickles originate from? and (iii) How do the prickles develop? We clearly demonstrated that prickles in rose originate from the ground meristem and are not modified trichomes. Two major types of prickles are described in rose: glandular and nonglandular prickles. These histological analyses are necessary for precise genetic and genomic studies.

## Results

First, we performed a detailed analysis on individuals of a F1 progeny (macroscopic and microscopic analyses). Then, based on these observations, we performed a survey of prickle diversity in the genus *Rosa*, with more precise observations of twelve representative genotypes.

### Prickle type determination and anatomical study in the OW population

OW population obtained from the cross between *Rosa chinensis* ‘Old Blush’ (OB) and hybrid of *Rosa wichurana* (W), and both parents present prickles on their stems. Very clear separation of prickle traits (type and density) on stems was observed in the F1 hybrids. Based on the macroscopic analysis, we previously determined two categories of prickles on the stems of the OW progeny: (i) “nonglandular prickles (NGPs)” and (ii) “glandular prickles (GPs)” refer to the prickles without and with glands, respectively^28^. In addition, “prickless” refers to stems without prickles. For detailed morphological and anatomical studies of NGPs and GPs, we selected one individual presenting the two types of prickles, OW9106, and one without prickles, OW9068. According to the specific morphogenetic events during prickle development, we defined developmental stages for NGPs and GPs on the rose stem (as defined in ref. ^28^).

**Fig. 1:**
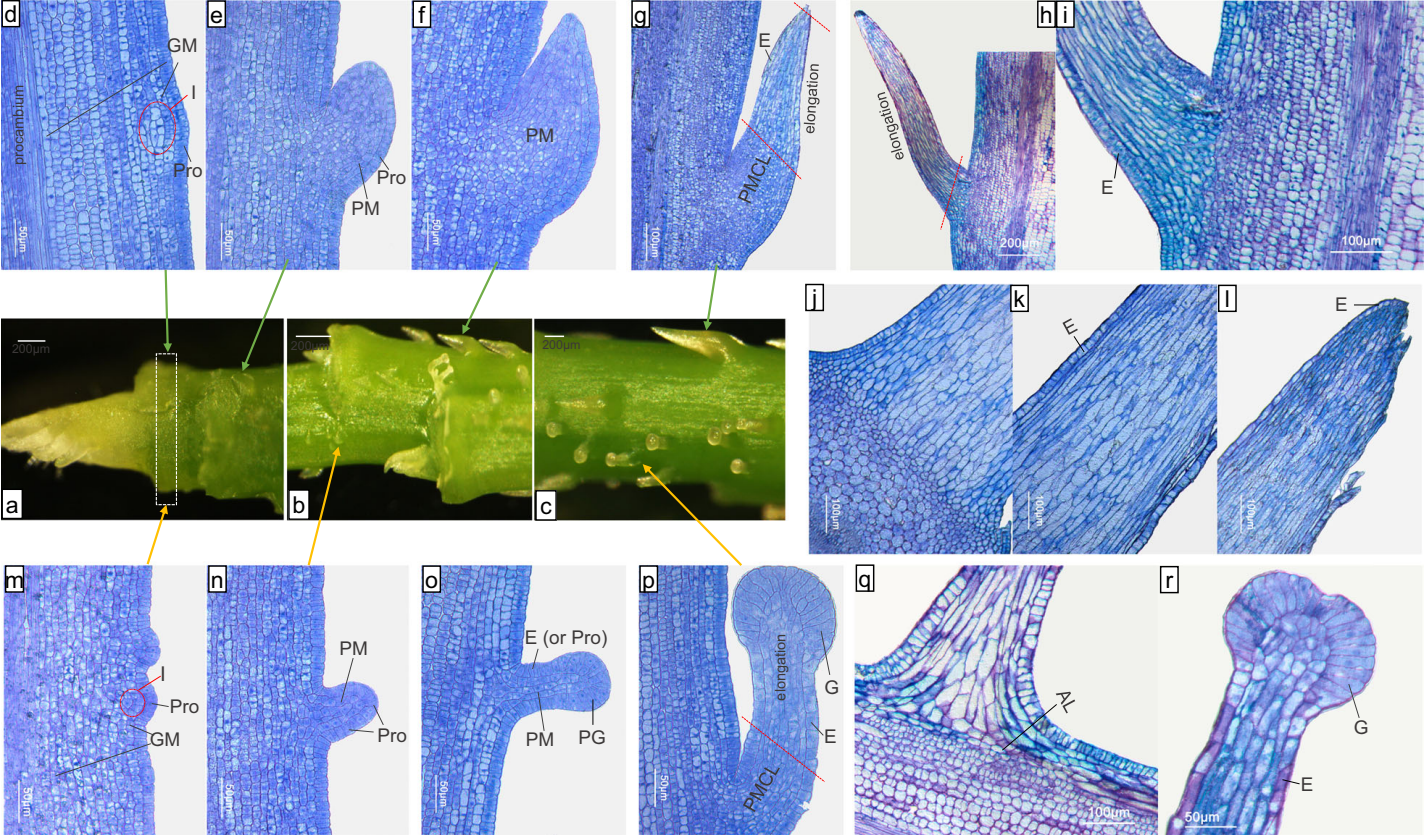
Anatomy of nonglandular prickles (NGPs) and glandular prickles (GPs) in OW9106. **a**–**c** Macroscopic images of the different stages of GPs and NGPs on the stem (leaves and leaf primordia were removed). Anatomy of stage I (**d**–**f**), IIa (**g**), IIb (**h**, **i**) and IIc (**j**–**l**) of nonglandular prickles. Anatomy of stage I (**m**–**o**), IIa (**p**), and III (**q**, **r**) of glandular prickles. The white dotted frame represents the first internode. I: prickle initiation; Pro: protoderm; GM: ground meristem; PM: prickle meristem; PMCL: prickle meristematic cell-like; E: epidermis; PG: precursor gland; G: gland; AL: abscission layer structure-like

**Fig. 2:**
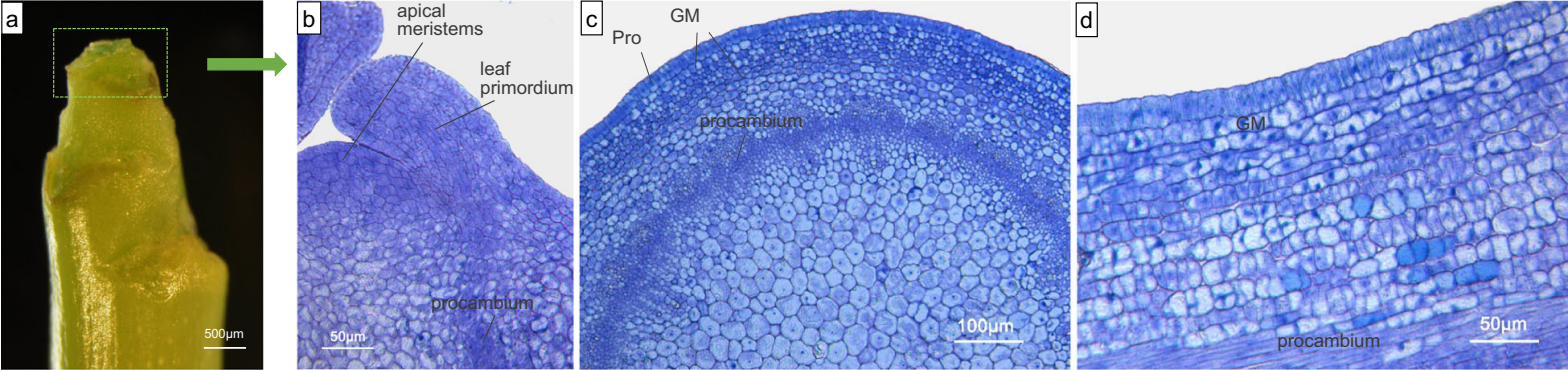
Anatomy of prickless stems in OW9068. **a** Macroscopic image of a prickless shoot tip (leaves and leaf primordia were removed). **b** Longitudinal sections of shoot tips. **c**, **d** Cross- and longitudinal sections below the apical meristems, respectively. GM: ground meristem. Pro: protoderm. E: epidermis

Stage I corresponds to prickle initiation and to the first outgrowth. Initiation appeared at the early stage of internode development (probably simultaneously with the first internode, under the petiole (Fig. 1a, white dotted frame)). It appeared just below the formation of leaf primordium. The first visible sign of an NGP was proliferation of multiple cells of the ground meristem (Fig. 1d). The rapid division of those cells causes an oblique rise leading to a triangular protuberance (100–500 µm), which can be observed on the macroscope (Fig. 1a, d). This process was absent in the prickless OW9068 genotype: no appendages were observed (Fig. 2). For GPs, 2–4 cells located at the first (and/or second) layers of the ground meristem first appeared to differentiate and to divide (Fig. 1m), and they gave rise to a cylindrical bump (~50 µm) (Fig. 1n). At this early stage, a difference was observed between GPs and NGPs of OW9106 in the number of primordial cells (Fig. 1d, m). The GP primordia are smaller than the NGP ones. Then, in GPs and NGPs, the rapid cell division of a limited region of the ground meristem gives rise to a new structure on the surface of the stem (Fig. 1e, n). For GPs, the protoderm of this new structure differentiates into precursor gland cells (Fig. 1o), which will give rise to gland cells. This differentiation is absent in NGPs, where the protoderm (or the epidermis) only continues to grow by cell division (Fig. 1f).

In Stage II, both NGPs and GPs show continuous growth, color development, and shape development (Supplementary Fig. 1 presented in Zhou et al.^28^). The difference between GPs and NGPs is that the precursor gland cells of GPs form a new structure—the glandular head—whereas no such structure is observed at the tip of the NGPs (Fig. 1g, p). We have divided stage II into three substages:

For NGPs, epidermal cells maintain normal cell proliferation during prickle development (Fig. 1g–l). In stage IIa, prickles continue to grow upwards^28^ because the young prickle is covered by unopened leaves. Anatomical analysis showed that the upper cells (from up to down) of the prickle begin to enlarge, suggesting that the cells gradually lose their ability to divide, while the cells of the lower part may still continue to divide (small cells) (Fig. 1g). These cell proliferation abilities and cell division orientations may determine the prickle shape and the width of the prickle base in the later stages. In stage IIb, as the leaves open, the prickles grow outwards^28^. The cells of the lower half of the prickle gradually stop proliferating and begin to elongate (from top to bottom, Fig. 1h, i). In stage IIc, after the leaves are fully opened, the prickles are almost fully developed and form downwardly curved hooks^28^. All the cells gradually stop dividing and continue to elongate lengthwise (Fig. 1j–l).

The developmental stages of GPs are similar to those of NGPs, except for the development of the gland head (Fig. 1p; ref. ^28^). The gland is usually surrounded by one cell layer and occasionally by two cell layers (Fig. 1p). Cell division stops at early stage IIa. Then, the cells only enlarge, leading to the formation of a glandular head (Fig. 1p). Their size only slightly increases during GP development (100–150 µm).

The NGPs and GPs enter stage III when they begin to lignify and gradually harden^28^. An abscission layer structure-like is also formed (Fig. 1q). Thus, the prickles can be easily separated from the stem. At the end of this stage, the cells are fully enlarged and lignified.

Stage IV is defined as the mature stage, in which the NGPs and GPs completely harden, lose moisture and exhibit gradual cell death^28^.

### Discovery of different types of prickles among the rose resources

**Fig. 3:**
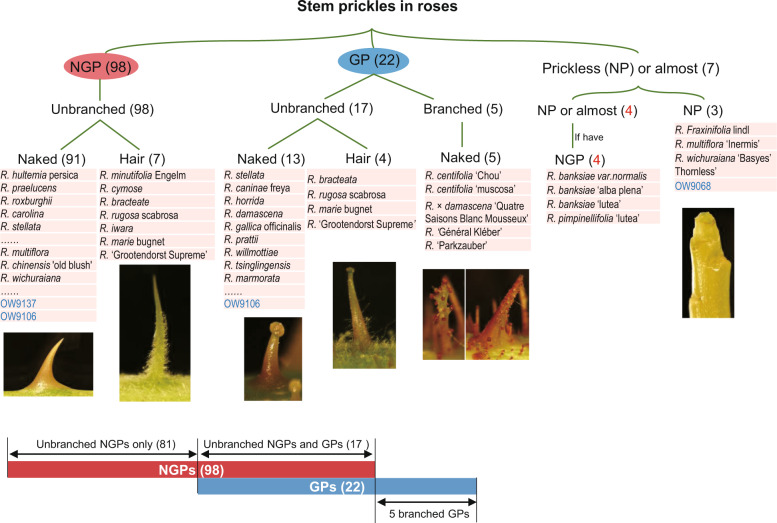
Different types of prickles among the rose resources. The number of roses is presented in the brackets. The “4” in red means that four prickless genotypes can sometimes present a few NGPs (*R. banksiae var. normalis*, *R. banksiae* ‘alba plena’, *R. banksiae* ‘lutea’, and *R. pimpinellifolia* ‘lutea’). OW F1 individuals (shown in blue) are not counted in the number of each category indicated in the figure. Ninety-eight roses present NGPs, including 81 roses presenting NGPs only and 17 presenting NGPs and GPs simultaneously (as in OW9106)

To describe the different types of prickles that are present in the *Rosa* genus, we conducted a survey of prickle types in 110 wild rose species, varieties, and ancient hybrids (Supplementary Table 1, Fig. 3). Twelve representative individuals (highlighted in pink in Supplementary Table 1), which represent different sections of the *Rosa* classification, were selected for detailed morphological analysis. According to macroscopic observations, we classified prickles into two general categories, glandular prickles (GPs) and nonglandular prickles (NGPs), as we previously observed for OW individuals. The majority of roses present NGPs (98 out of 110), including 81 roses that presented NGPs only and 17 that presented NGPs and GPs simultaneously (as previously shown for OW9106) (Fig. 3). The NGPs and GPs in these 98 roses were all unbranched. The unbranched NGPs and GPs of a few roses (7 and 4, respectively) are covered with hairs (hairy), whereas the majority (91 and 13, respectively) did not have hair (naked). Five genotypes present only branched and naked GPs (Fig. 3, Supplementary Table 1). Seven roses have glabrous stem, but among these roses, four can sometimes be observed with a few NGPs. Here, we describe the characteristics and developmental process of the different types of prickles through detailed analysis of examples of rose resources.

### Unbranched NGPs

#### Naked

**Fig. 4:**
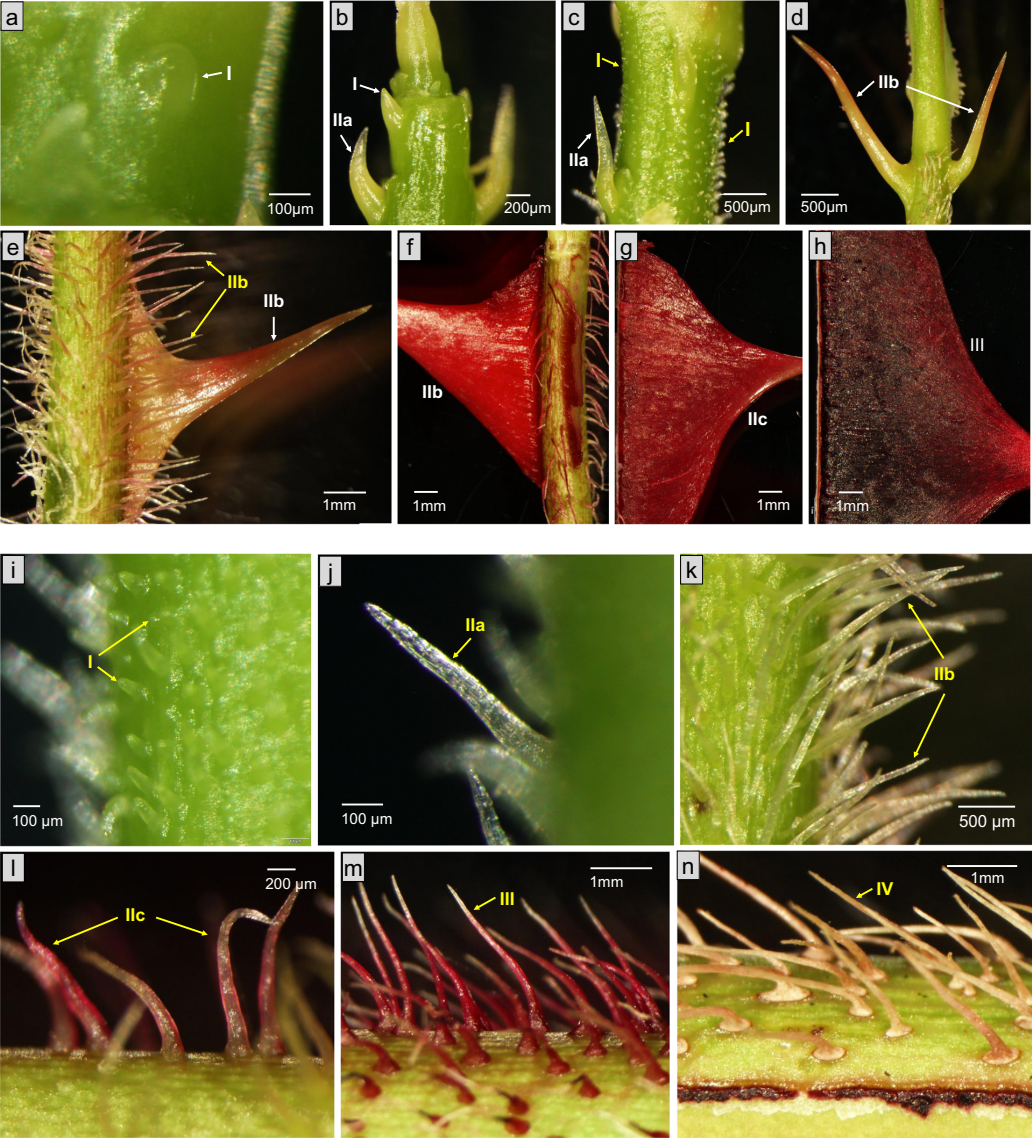
Nonglandular prickle developmental process in *R. omeiensis.* The initiation of needle-like prickles (**i**–**n**) occurs later than that of wing-like prickles (**a**–**h**). White and yellow alphabetic marks and arrows refer to the development stages of wing-like and needle-like prickles, respectively

The unbranched and naked NGPs (91 genotypes in our rose collection) are the most common type of prickles in our rose collection. Among the representative genotypes, five (*R. omeiensis, R. ecae, R. laxa, R. sherardii*, and *R. moschata*) present only naked NGPs. The mature prickles are highly diverse in terms of shape, color, size, and density (Fig. 4, Supplementary Figs. 2 and 3). In particular, there are two very different shapes of NGPs present in *R. omeiensis*, which are needle-like (bristles) and wing-like (Fig. 4a). We found that the morphology of stages I and IIa of the prickles (previously defined for OW9106) are similar in these species (except the bristle prickles, Fig. 4i–n). The primordial cells gave rise to an oblique triangular structure (100–500 µm) that grows upwards (Fig. 4a, b). A large difference in shape appears at the later stages. We also observed slight differences in prickle initiation in different genotypes. In *R. ecae* (Supplementary Fig. 2a–e), *R. laxa* (Supplementary Fig. 2f–i) and *R. omeiensis* (large wing-like prickle, Fig. 4a–h), prickle initiation occurred only at the shoot tip, and the same stage of prickles appeared in the same region of the stem. Their development is quite similar to that described for the OW9137 prickles^28^. In *R. sherardi* and *R. moschata*, prickle initiation occurred not only at the shoot tip but also later during stem growth (Supplementary Fig. 3b and i). In these two species, prickle initiation can take place over a longer period, and the prickles that initiate later are smaller at maturity (Supplementary Fig. 3). Thus, the time and place of prickle initiation are important factors that impact the size of prickles on the mature stem.

#### Hairy

Some unbranched NGPs are covered with hairs (trichomes). Only seven genotypes (7 out of 110) in our collection have this type of prickle (Fig. 3, Supplementary Table 1): *R. minutifolia* Engelm, *R. cymosa, R. bracteata, R. rugosa* ‘scabrosa’ (Supplementary Fig. 4a–g) and three hybrids of *R. rugosa* (*R. iwara* (Supplementary Fig. 4h–q), *R*. “Grootendorst Supreme” (Supplementary Fig. 4r–v) and *R*. ‘Marie bugnet’). In *R. rugosa* ‘scabrosa’, hairs are present on the stem and on the prickles. On the stem, high-density hairs are present all along the shoot. Their initiation occurred earlier than that of prickles. During prickle development, no hairs were visible in stage I (Supplementary Fig. 4a). Later, hairs appeared on the lower part of the prickle, and the upper part remains naked throughout development. In *R. iwara*, the hairs appeared later and at a lower density (Supplementary Fig. 4k). Prickles and stems have no hairs during stages I to IIb (Supplementary Fig. 4h, i and m), and the hairs appear clearly at stages IIc and III (Supplementary Fig. 4k and l).

### Unbranched GPs

Seventeen roses in our collection present unbranched GPs, with 13 presenting naked GPs and 4 presenting hairy GPs (Supplementary Table 1, Fig. 3).

#### Naked

Concerning the unbranched and naked GPs, their developmental process and origin were described in the previous section. We found that these prickles always coexist with NGPs in roses (Supplementary Table 1), as in the following species or varieties: *R. iwara* (Supplementary Fig. 4n and o), *R. stellata*, *R. caninae* ‘freya’, *R. horrida*, *R. rubella* (Supplementary Fig. 5a–f), *R. damascena* (Supplementary Fig. 5g–j), *R. gallica officinalis*, *R. prattii, R. willmottiae, R. tsinglingensis, R. marmorata, R. pimpinellifolia* ‘King of the Scots’, *R. pimpinellifolia* ‘aïcha’ and *R. anemoniflora*.

#### Hairy

Unbranched GPs are covered with hairs (trichomes). Only four genotypes have this type of prickle: *R. bracteata*, *R. rugosa* ‘scabrosa’, *R*. ‘Marie Bugnet’ and *R*. ‘Grootendorst Supreme’ (two hybrids of *R. rugosa*) (Supplementary Fig. 4t and u). They also present hairy NGPs on their stems.

### Branched GPs

**Fig. 5:**
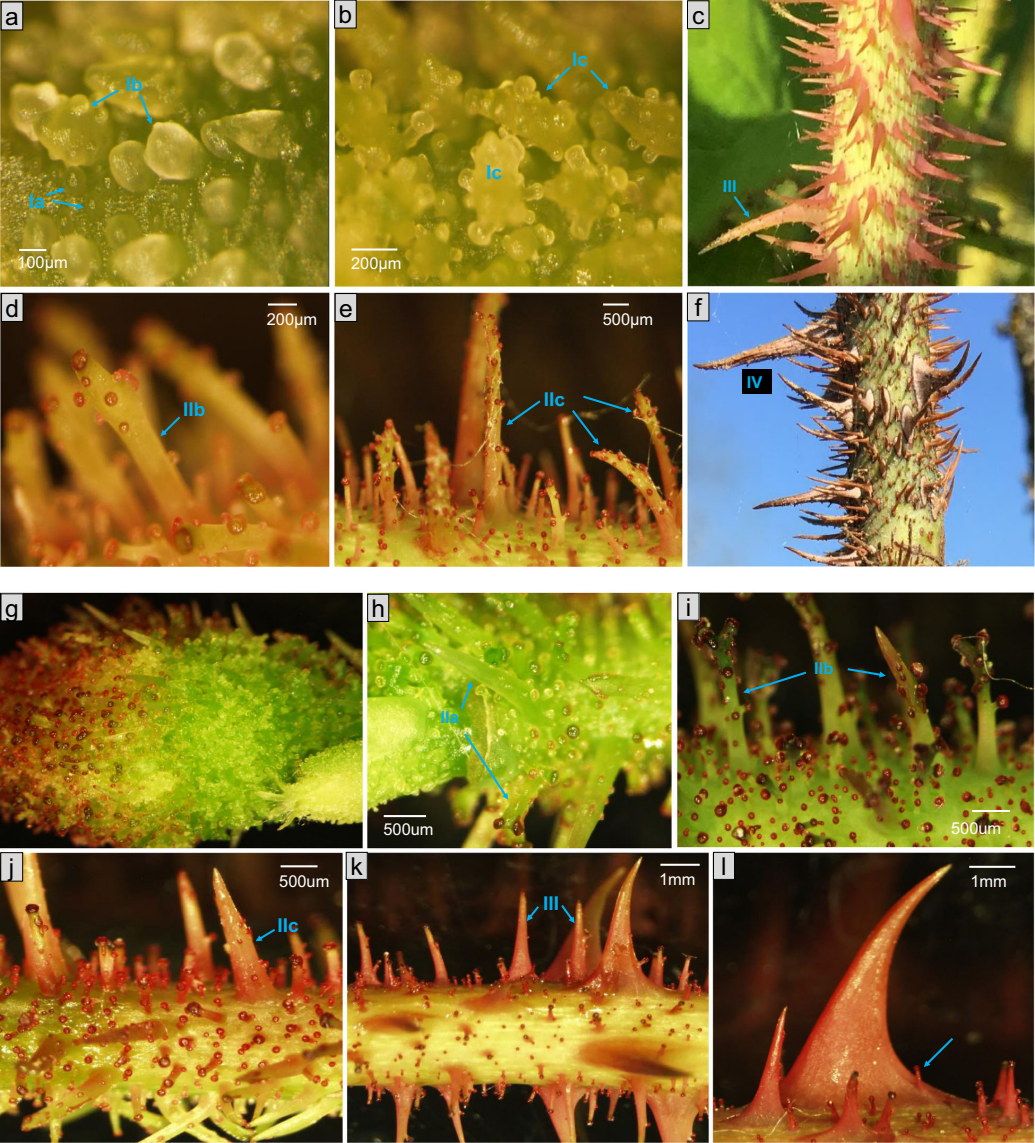
Branched and naked glandular prickle development in R. ‘General Kleber’ and R. ‘Parkzauber’. Stage Ia, Ib (**a**), Ic (**b**), IIb (**d**), IIc (**e**), III (**c**) and IV (**f**) of GPs in *R*. ‘General Kleber’. **g** Early stage of GPs on bud of *R*. ‘Parkzauber’. Stage IIa (**h**), IIb (**i**), IIc (**j**), III (**k**) of GPs in *R*. ‘Parkzauber’. **l** A small GP developed on a large NGP

Branched GPs were found in only five roses: *R. centifolia* ‘chou’, *R. centifolia* ‘muscosa’, *R. × damascena* ‘Quatre Saisons Blanc Mousseux’, and the two hybrids *R*. ‘Général Kléber’ (Fig. 5a–f) and *R*. ‘Parkzauber’ (Fig. 5g–l). All the prickles were naked, and no hairy types were found in this subcategory.

Interestingly, these roses belong to a particular type of roses, the moss roses (see “Discussion”). At stage I, the developmental process of branched GPs is more complicated than the one of unbranched GPs. Thus, we divided stage I into three substages. In stage Ia, multiple divisions give rise to a nearly round protuberance (Fig. 5a). The appearance of branch bumps is a sign of entrance into stage Ib (Fig. 5a). In stage Ic, the bumps continue to grow and to differentiate into glands and stalks (Fig. 5b). The subsequent stages are similar to those of the unbranched GPs (Fig. 5c–f).

Some GPs present only one gland head (Supplementary Figs. 4 and 5), whereas some have several glands randomly distributed on their surface (Fig. 5). Some genotypes are not easy to classify in the previous categories. For example, one or several small GPs can develop on a large NGP (Fig. 5l), as observed in *R*. ‘General Kleber’.

All prickles go through initiation, development, and senescence. Most prickles do not fall from the stem, but a few do (Supplementary Fig. 3g). In such cases, only a scar is visible.

## Discussion

### Two types of prickles in roses, glandular and nonglandular, with two distinct gene networks

On the basis of the morphology and anatomy of prickles, from their initiation to their complete development, and their distribution in the OW population, we proposed a categorization scheme for the presence or absence of prickles in roses. We defined two major types of prickles in roses: nonglandular prickles (NGPs) and glandular prickles (GPs).

For the first time, NGP and GP initiation and development were histologically characterized in detail. At the initiation stage, no essential difference was observed between GPs and NGPs. Both arose from the ground meristem beneath the protoderm. Later, differences appeared between NGPs and GPs. GPs rapidly developed a gland that was absent in NGPs. Our results do not support previous research showing that GPs and NGPs are the early and later stages of the same prickle^2,5,22,26^. Furthermore, our conclusions are different from those of previous studies, which reported that prickles originated from the epidermis^5,24,25^ and were modified from glandular trichomes^22,26^, were induced from glandular trichome signals^40^, or originated from bark tissue^13^. The sub-epidermal origin of prickle in rose was confirmed in a recent study for the cultivar “First Red”, where prickles are proposed to be originated from the cortical parenchyma^30^.

We suggest that GPs may be modified from NGPs for the following reasons. NGPs are the most common type of prickles in roses, and GPs (except for a few genotypes with branched GPs) always coexist with NGPs. Furthermore, GPs and NGPs have a common initiation process, and their development differs later (Fig. 1). For GPs, gland cells (a specific structure of GPs) are not produced at prickle initiation but during prickle development (Fig. 1m–r). A similar hypothesis was proposed for the trichomes. From an evolutionary perspective, the earliest glandular trichomes (GTs) were proposed to be modified from nonglandular trichomes (NGTs)^41,42^. Another possibility is that GPs and NGPs have different genetic pathways in terms of the fate of the first mother cells. Additional studies are needed to test these different hypotheses. The genetic results support the last hypothesis^28^.

At the morphological level, we also observed a large diversity of GPs and NGPs in the *Rosa* genus. The glandular and nonglandular prickles can be covered by hairs (hairy) or not (naked). GPs can present branches (branched versus unbranched GPs). For hairy prickles, hair initiation occurs later than prickle initiation, and these hairs cover not only the prickles but also the stem (Supplementary Fig. 4). Therefore, we hypothesize that: the genetic pathways for hairy and naked NGP initiation are the same; the genetic mechanism controlling hair initiation is different from that controlling prickle initiation. Similarly, branches appear during prickle development, suggesting that the pathways controlling GP initiation are similar between unbranched and branched GPs, whereas another signaling pathway might control whether a prickle branches or not.

### Suggestions for genetic and genomic studies on rose prickles

As we suggested for the first time that different genetic pathways are involved in GP and NGP initiation^28^, these pathways should be studied separately in genetic and molecular studies. In roses, prickles are present on organs other than the stem, such as petioles, pedicels, and fruits. Here, we focused on the presence of prickles on the stem. According to the specific morphogenetic events during their initiation and development, we divided the development of unbranched NGPs and GPs into four stages, with three substages for stage II. For branched GPs, we divided stage I into three substages. The stages of NGPs have been used for reverse genetics^28^, and the stages of different types of prickles are key resources for detailed transcriptomic analyses.

Quantitative trait locus (QTL) analysis and genome-wide association studies (GWASs) are the most popular methods used to reveal the genetic bases of quantitative traits. Both methods are achieved by looking for a correlation between phenotype and genotype. Therefore, how to evaluate the phenotype is an important question for starting these two genetic approaches. Here, we propose a rapid way to phenotype the different prickles in roses according to a few criteria (Fig. 3): first, the presence of a glandular head (GPs vs. NGPs); then, the presence of branching (unbranched vs. branched prickles); and finally, the presence of hairs on the prickles (naked vs. hairy). This new phenotyping method can be used to phenotype prickles in genome-wide association panels, where a large diversity of prickles can be present.

### Prickless roses may be linked to human selection

We noticed that glabrous roses were rare among our 110 samples: only 7 glabrous roses (bearing no GPs or NGPs) were found. Among them, four were sometimes observed with a few prickles, and one (*R. fraxinifolia* Lindl) was described as occasionally bearing a few prickles (during our scoring, no prickles were observed)^43^. The majority of glabrous roses are in fact cultivars selected from wild species: *R. multiflora* ‘inermis’, *R. wichurana* ‘Bayses’ Thornless’, *R. pimpinellifolia* ‘Lutea’, *R. banksiae* ‘Alba Plena’ and *R. banksiae* ‘Lutea’. Only two wild species (*R. banksiae var. normalis* and *R. fraxinifolia* Lindl) were glabrous. This suggests that glabrousness is not adaptive in the wild, and there might be selection pressure on roses to maintain prickles under natural conditions. The absence of prickles may be due to mutations, and selection may eliminate this trait, similar to selection for recurrent blooming trait^44^. Therefore, our hypothesis is that glabrous mutants were selected and rescued by humans. This could explain the rarity of genotypes without prickles found under natural conditions. These glabrous roses are interesting materials for genetic and genomic studies aiming to understand prickle initiation.

### Branched GPs in moss roses

Branched GPs are also rare and were present in only 5 of the 110 roses examined here: *R. centifolia* ‘Chou’ (Inconnu, <1595), *R. centifolia* ‘muscosa’ (<1700), *R. × damascena* ‘Quatre Saisons Blanc Mousseux’, *R*. ‘Général Kléber’ and *R*. ‘Parkzauber’ (1956)^45^. Interestingly, these roses are all moss roses. The flower pedicels and calyxes of moss roses are covered with moss-like growths (they may be glandular trichomes or emergences). Moss roses are old garden roses belonging to the subgenus *Rosa* sect. *Caninae* DC^43^. *R. × centifolia* ‘muscosa’ may be obtained by bud mutation. *R. × damascena* ‘Quatre Saisons Blanc Mousseux’ may be a sport or bud mutation of *R. × damascena* ‘bifera’, which is a repeatedly blooming hybrid of *R. × damascena*^46^. *R. × damascena* ‘Quatre Saisons Blanc Mousseux’ was the first repeatedly blooming cultivar among moss roses^46^. The exact genetic relationship between *R. × damascena* and *R. centifolia* is still unclear. The origin of the moss roses is also unknown. *R. × damascena* presents only unbranched GPs and NGPs on the stem and leaves, suggesting that branched GPs on the stem may be obtained from *R. × centifolia* but not *R. × damascena*.

### Old questions and new insights: prickles and trichomes

Prickles and trichomes show a certain correlation and degree of resemblance. First, both trichomes and prickles have nonglandular and glandular as well as branched and unbranched forms. Both lack vascular bundles. Second, from functional and herbivore coevolutionary perspectives, prickles and trichomes may have a certain correlation. For example, some trichomes and prickles are associated with adaptation to drought conditions and protection against herbivories (especially insects) (review by Zhou^17^). Combining morphological, ultrastructural, chemical, and molecular evidence could help decipher the common features between prickles and trichomes.

However, important distinctions exist between trichomes and prickles. Trichomes are epidermal appendages that originate from one or more protoderm (or epidermis) cells only^10,15^. In this work, we showed that prickles originate from the tissue beneath the protoderm, which we called the ground meristem (Fig. 1). Usually, cells at different positions perceive different signals, respond through intracellular signaling pathways and eventually adopt a specific cell fate, thereby producing different organs or tissues^47^. Therefore, for trichomes and prickles, the tissues they originate from are different (protoderm versus subprotoderm), which may indicate that different gene networks control prickle and trichome initiation. This hypothesis is supported by molecular evidence in roses, where no strong link can be found between the trichome and prickle pathways. Indeed, Zhou et al.^28^ characterized rose gene homologs known in *A. thaliana* to control trichome initiation. These genes were not transcriptionally regulated during prickle initiation, suggesting that the genetic pathway controlling prickle initiation is different from that controlling trichome initiation. Therefore, we suggest that different genetic pathways control the initiation of NGPs and NGTs. This conclusion is different from the current hypothesis: rose NGPs and *A. thaliana* unicellular NGTs share the same genetic pathway for their initiation^30,31^.

## Materials and methods

### Plant materials

A diploid OW population obtained from a cross between *Rosa chinensis* ‘Old Blush’ (OB) and *Rosa × wichurana* (RW) was grown in a field and managed by the Horticulture Experimental Unit (INRAE, Angers, France). To obtain more vegetative branches, we selected two once-flowering individuals OW9068 and OW9106. These genotypes were cut and managed in IRHS greenhouses in November 2017.

*Rosa* resources were planted at the Loubert Rose Gardens (Rosiers sur Loire, France), INRAE (Angers, France) and Flower Research Institute (FRI, Kunming, China). We selected twelve representative genotypes to perform detailed analyses of the type and developmental stages of prickles: *Rosa ecae*, *Rosa laxa*, *Rosa sherardi*, *Rosa moschata*, *Rosa omeiensis*, *Rosa damascena*, *Rosa rugosa* ‘scabrosa’, *Rosa iwara*, *Rosa* ‘Grootendorst Supreme’, *Rosa rubella*, *Rosa* ‘General Kleber’ and *Rosa* ‘Parkzauber’. For 110 genotypes (Supplementary Table 1), we scored the prickle type on the first and second branches, and only considered prickles on the stem (prickles on pedicel were excluded).

### Macroscopy and stereoscopy

The experiments were performed at Platform IMAC (SFR QuaSav, Angers). Fresh rose stems were photographed with a Leica M205FA stereomicroscope.

### Histological study

Sample dissections were performed under a microscope to remove the leaves. Various steps were performed:

- *Fixation at 4 °C*: Samples were immersed in a 4% (v/v) glutaraldehyde solution mixed with 0.2 mol/L phosphate buffer at pH 7.2. The solution volume was equal to 50 times the volume of the sample. Each sample was put under vacuum to remove air, with the vacuum setting paused every 4 min. After 2 h of vacuum, we changed the glutaraldehyde solution (4% v/v), stored the tubes at 4 °C 12 h, rinsed the samples twice with phosphate buffer pH 7.2 and stored the samples at 4 °C.
- *Dehydration at room temperature*: Samples were rinsed 3 times with distilled water and immersed in 50% (v/v) alcohol for 10 min, 70% (v/v) alcohol for 10 min, 90% (v/v) alcohol for 10 min, and 100% alcohol for 15 min.
- *Preinfiltration*: Samples were transferred to preinfiltration solution (100° alcohol/Technovit® 7100 resin (Heraeus Kulzer, Wehrheim, Germany) (v/v)) at 4 °C and under vacuum for 2 h, and then the samples were stored for 12 h at 4 °C.
- *Infiltration*: Samples were transferred to infiltration solution (1 sachet of hardener I dissolved in 100 mL (Heraeus Kulzer, Wehrheim, Germany) of Technovit® 7100 resin) under vacuum for at least 20 min at 4 °C, and the tubes were then stored for 12 h at 4 °C.
- *Inclusion*: Samples were included using an inclusion solution (1 mL of hardener II® (Heraeus Kulzer, Wehrhrim, Germany) and 15 ml of infiltration solution) and stored at 37 °C. Sections were made after a week at 37 °C.

The samples were cut into 3 µm sections for anatomical observation using a Leica RM2165 rotary microtome. After being stained with toluidine blue 1%^48^, the samples were observed and photographed using an ergonomic system microscope (Leica DM1000).

### Scoring of prickle type among the 110 roses

Taxonomical nomenclature followed that described in Yu^49^, Gu and Robertson^34^ and Masure^43^. Each species or hybrid was associated with the types of prickles that were previously determined in the OW population and in the twelve representative roses. The prickle types on each species were characterized based on photographs, which were mainly taken at the Loubert Rose Gardens (https://www.pepiniere-rosesloubert.com/) and FRI. For some species, the conclusions are based on professional knowledge and experience and online photographs. All the roses and their origins are presented in Supplementary Table 1.

## Supplementary information

The types of prickle on stem in the list of rose resources

General methods to distinguish trichomes, prickles, thorns, and spines

Non-glandular prickle developmental process in *R. ecae* (a-e) and *R. laxa* (f-i)

Non-glandular prickles developmental process in *R. sherardi* (a-g) and *R. moschata* synstylae (h-k)

Non-glandular and glandular prickles development in *R. rugosa* scabrosa (a-g), *R. iwara* (h-q) and *R.* ‘Grootendorst Supreme’(r-v)

Non-glandular and glandular prickle developmental process in *R. rubella* (a-f) and *R. damascena* (g-j)

## Data Availability

All data supporting the results of this study are included in the manuscript and its additional files.
