# Supplementary figures and images for: Morphological studies of rose prickles provide new insights
Source: Hortic Res. 2021 Sep 23;8:221. doi: 10.1038/s41438-021-00689-7 (PMC8460668; doi:10.1038/s41438-021-00689-7)

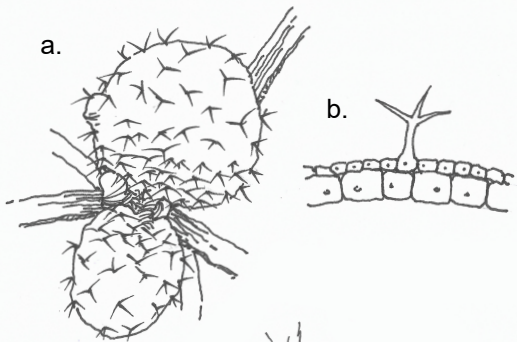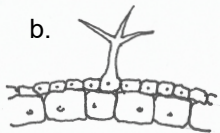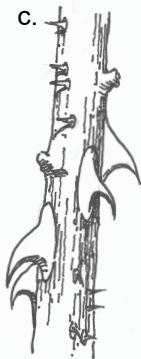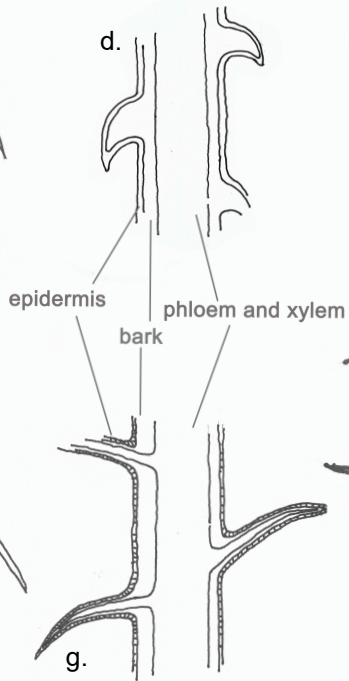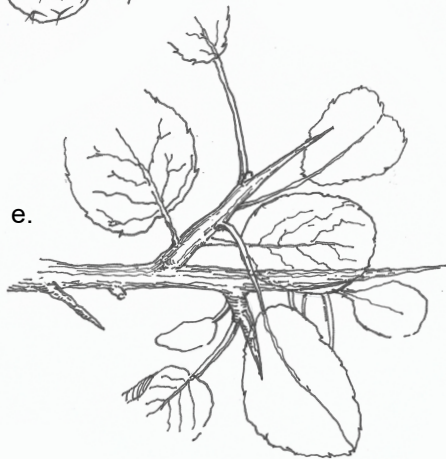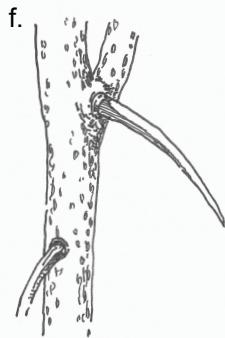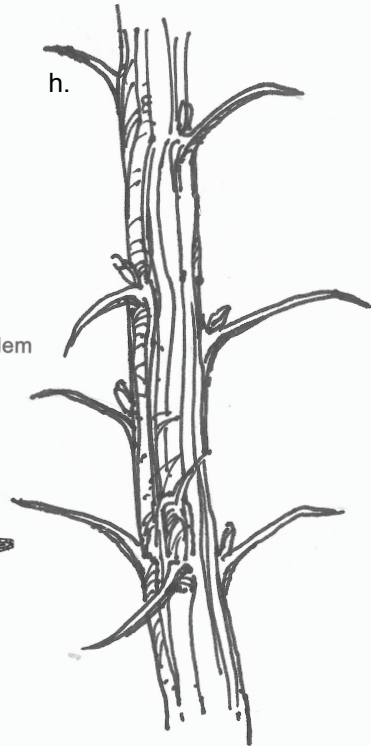

Supplement: Supplementary file 2 — General methods to distinguish trichomes, prickles, thorns, and spines [file 41438_2021_689_MOESM2_ESM.pdf]

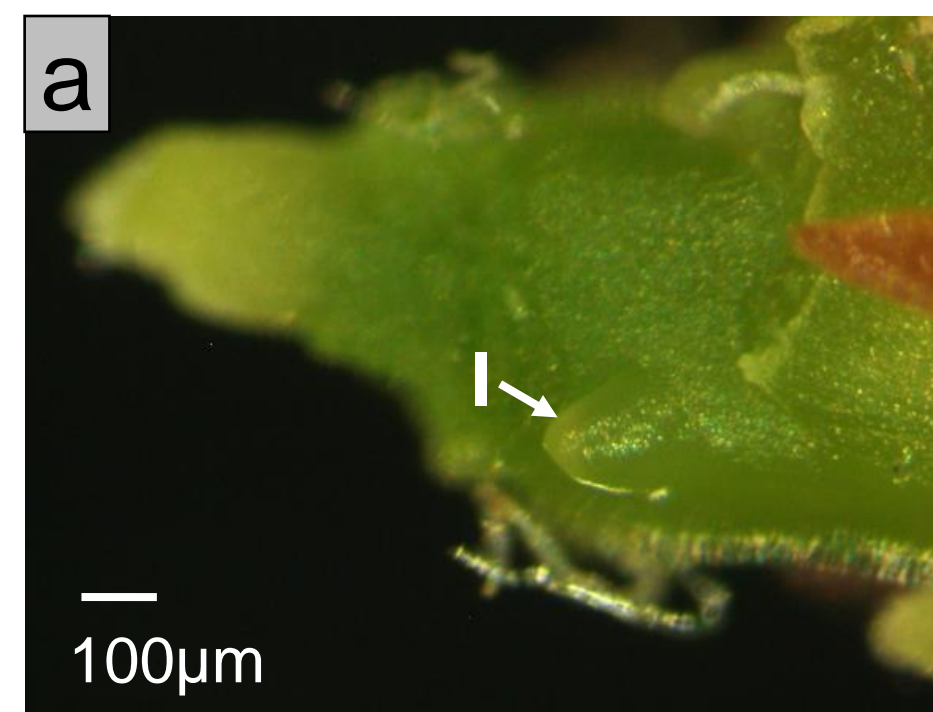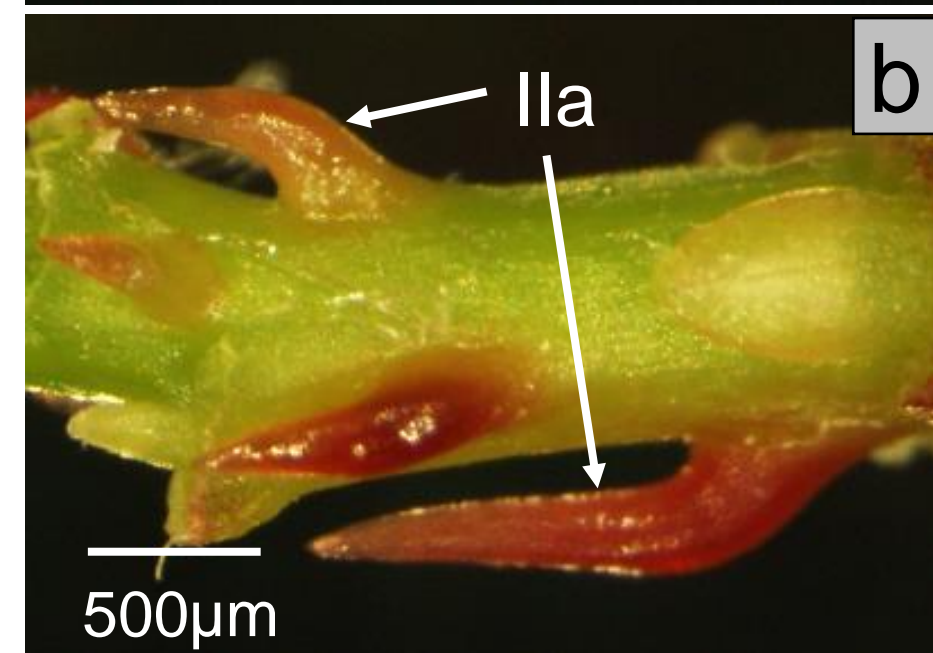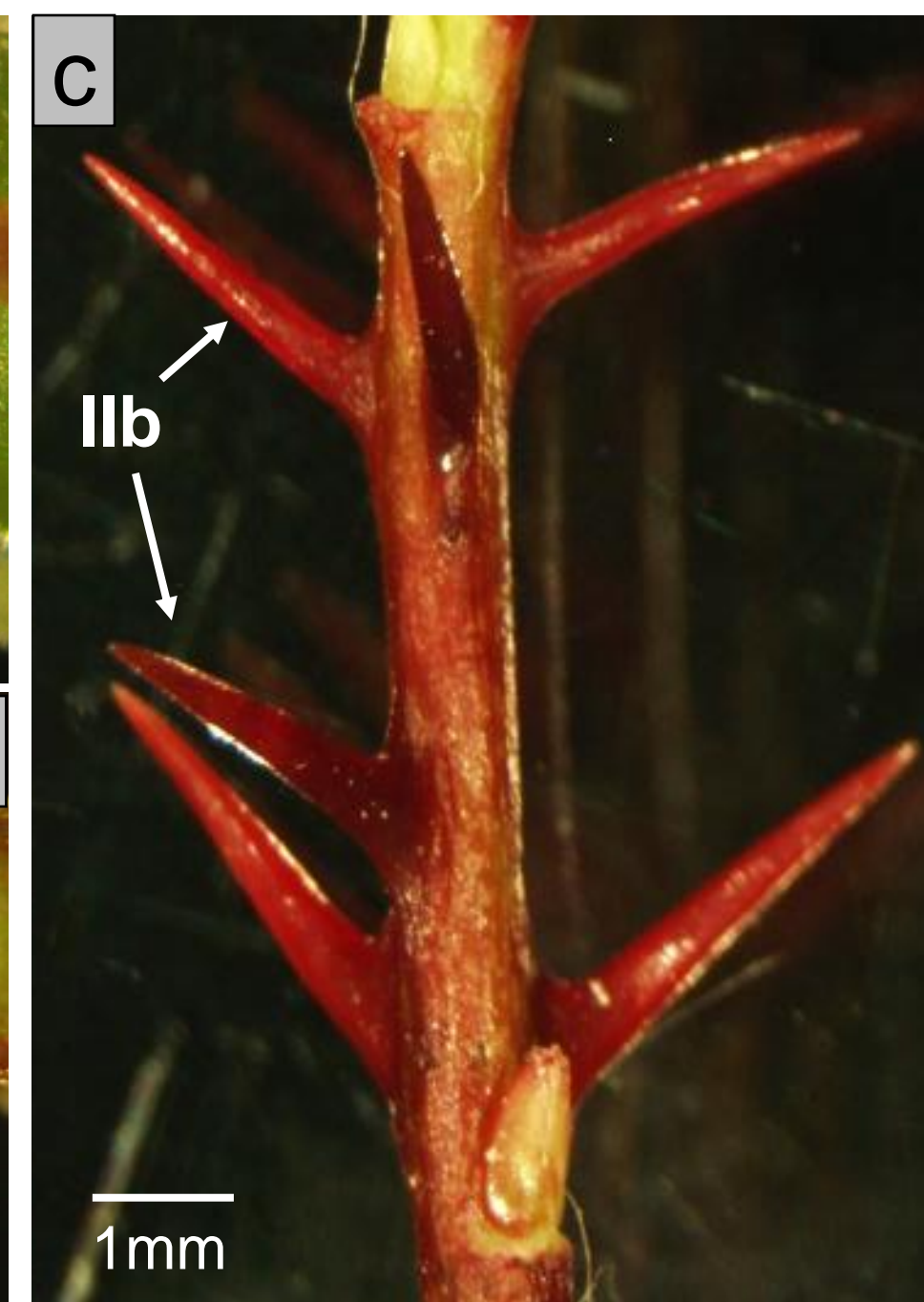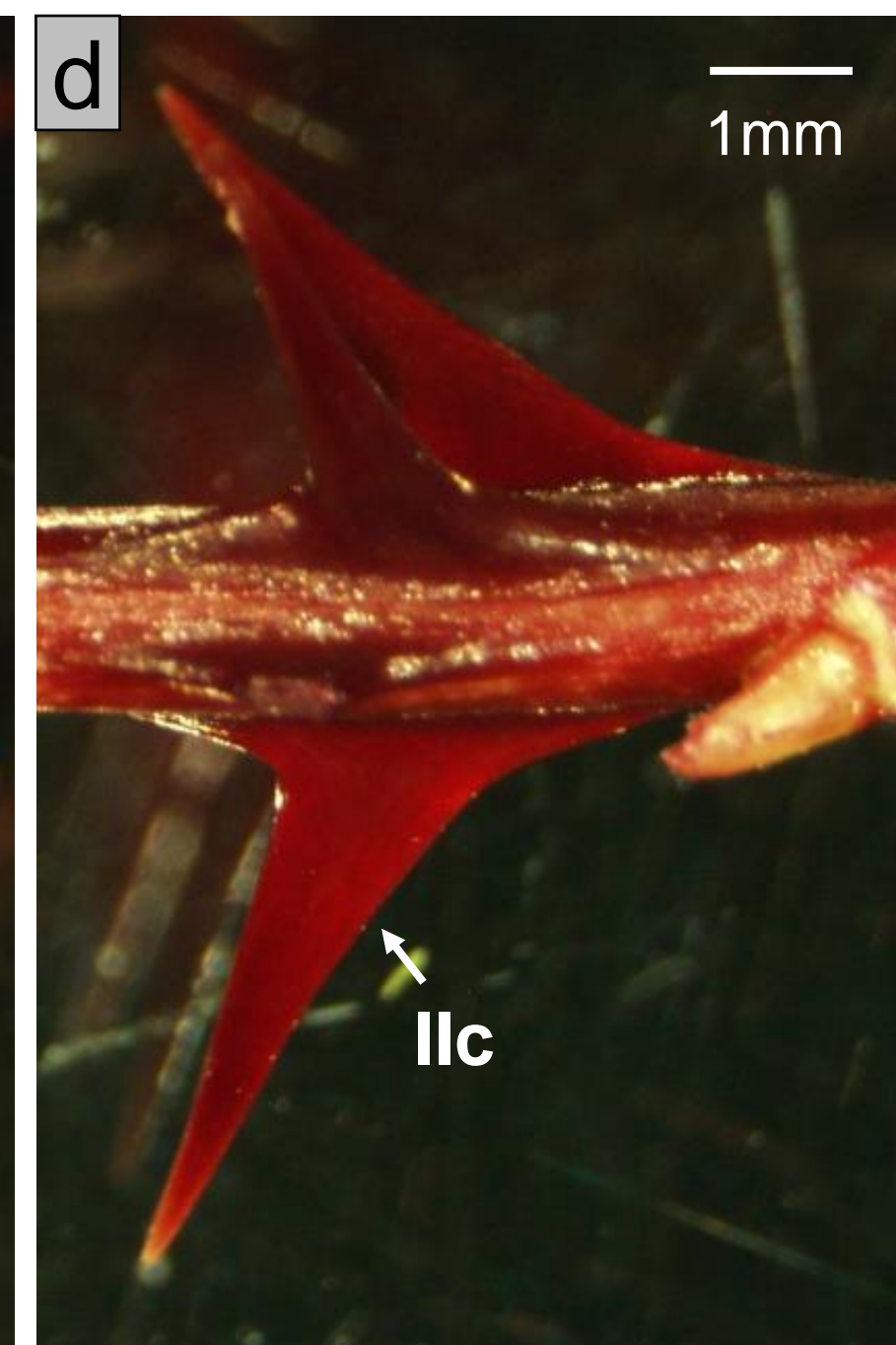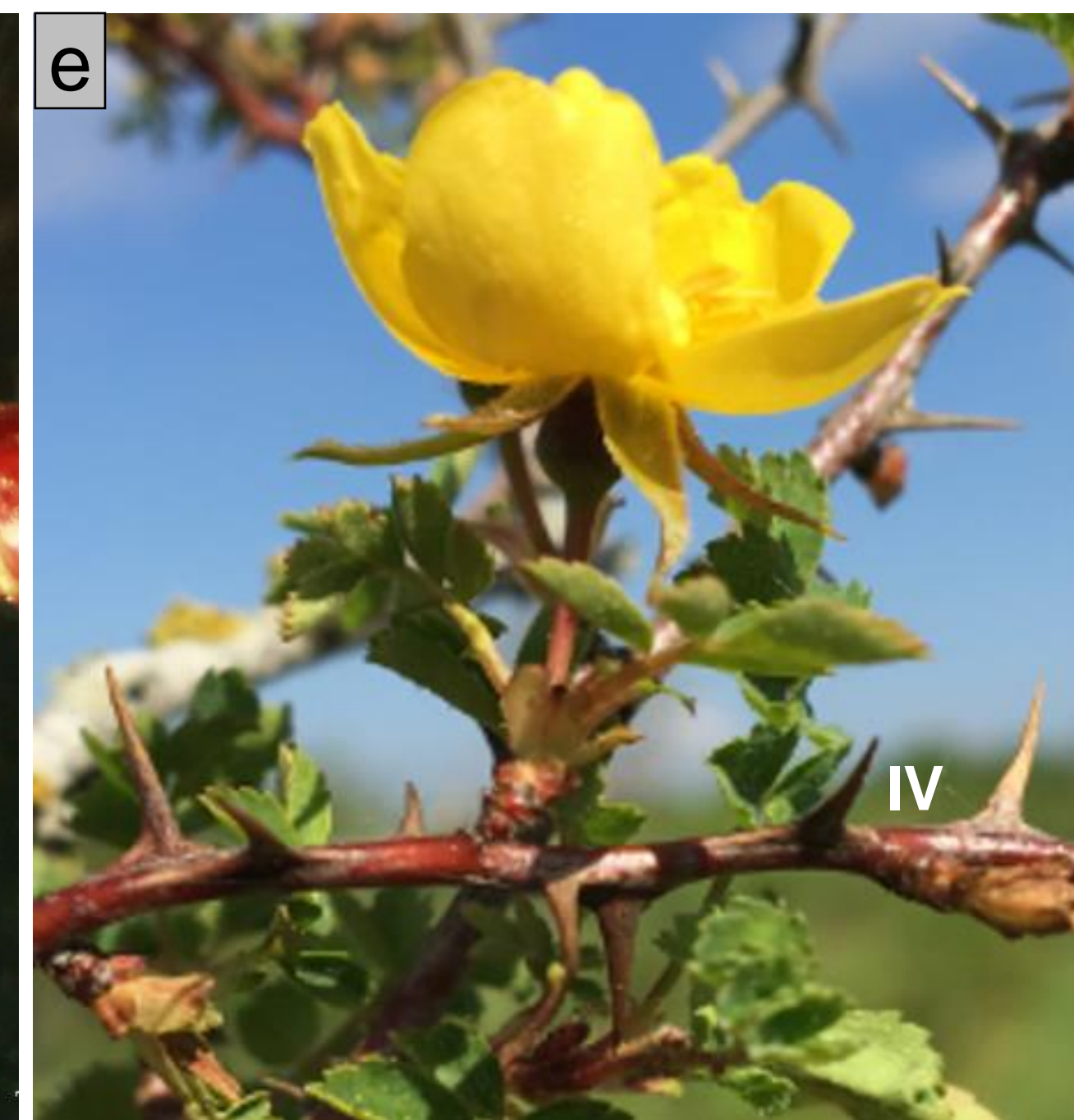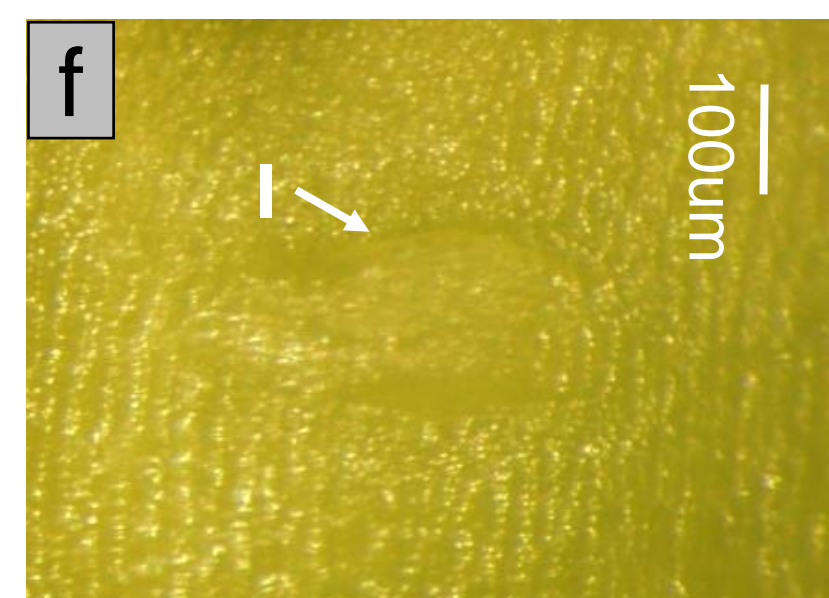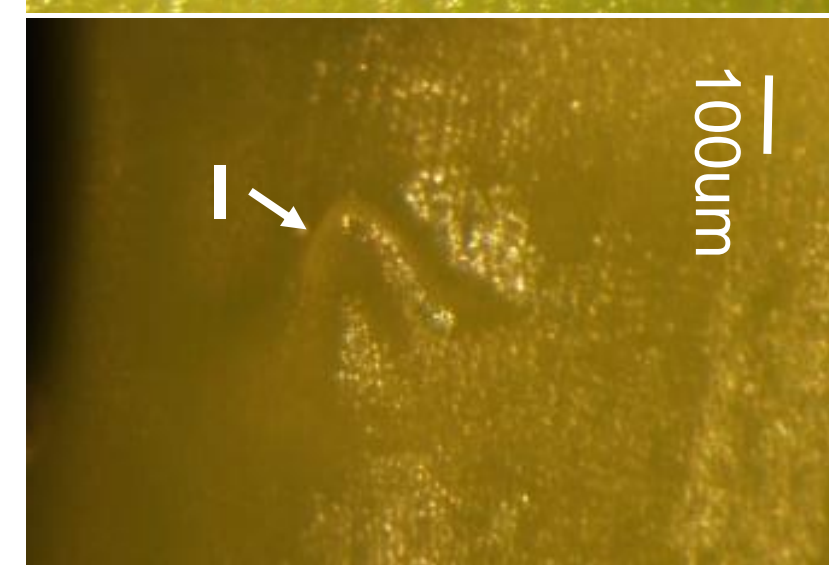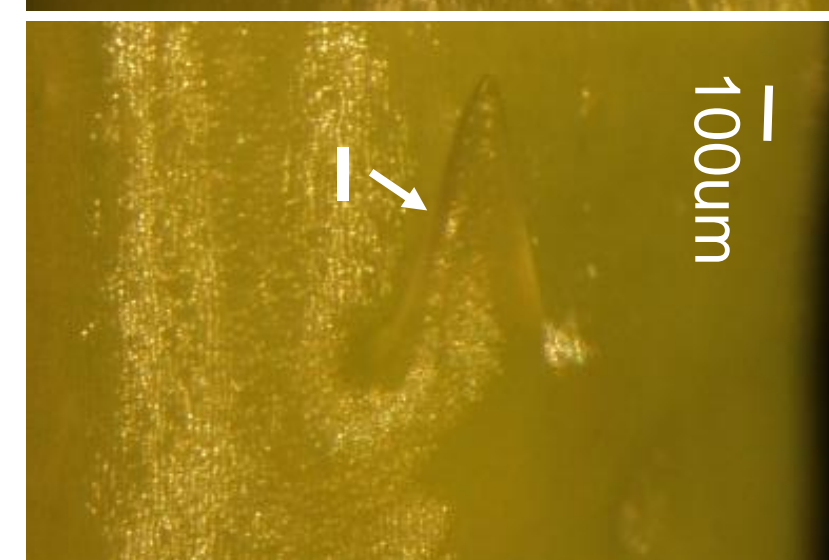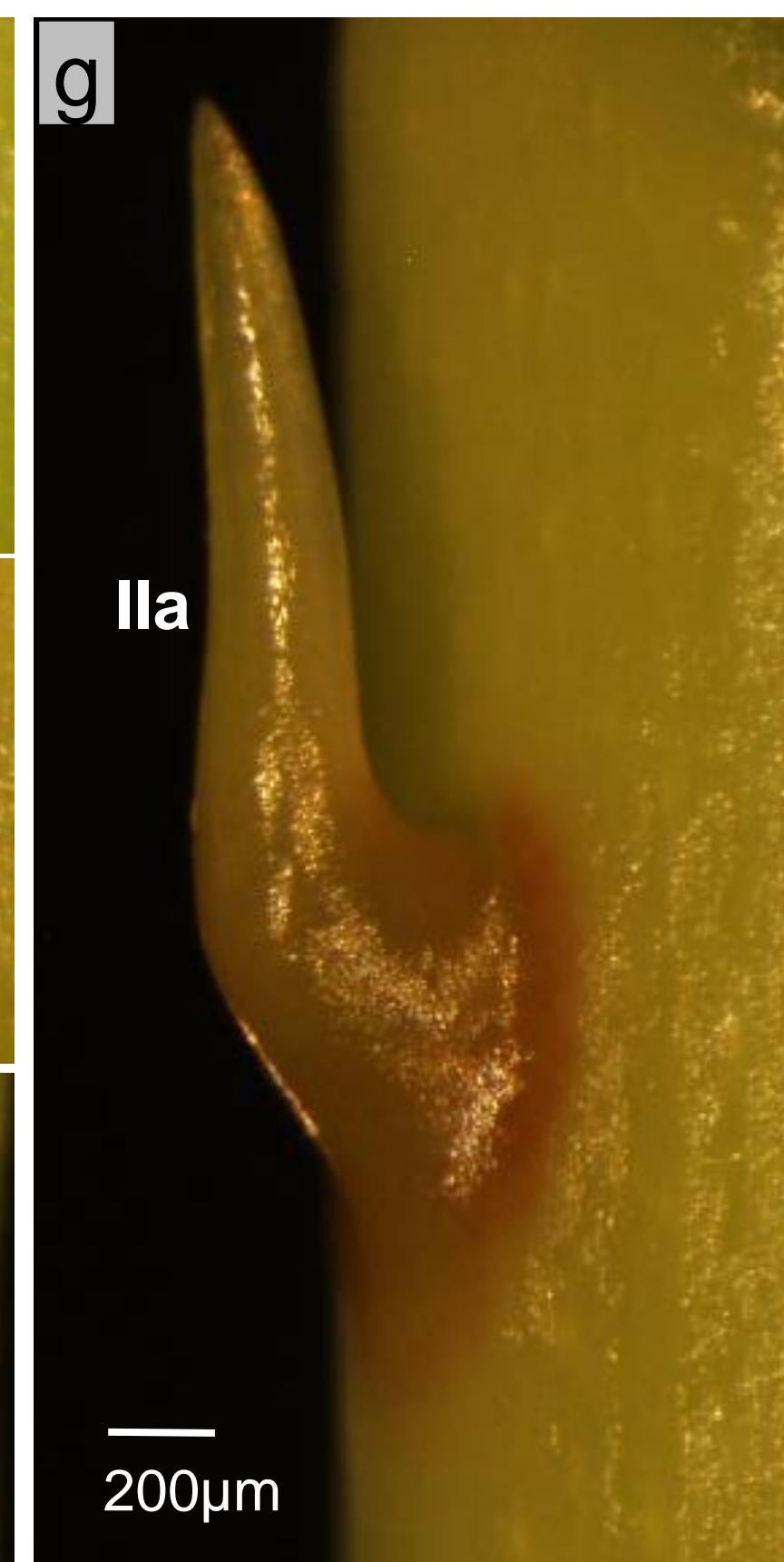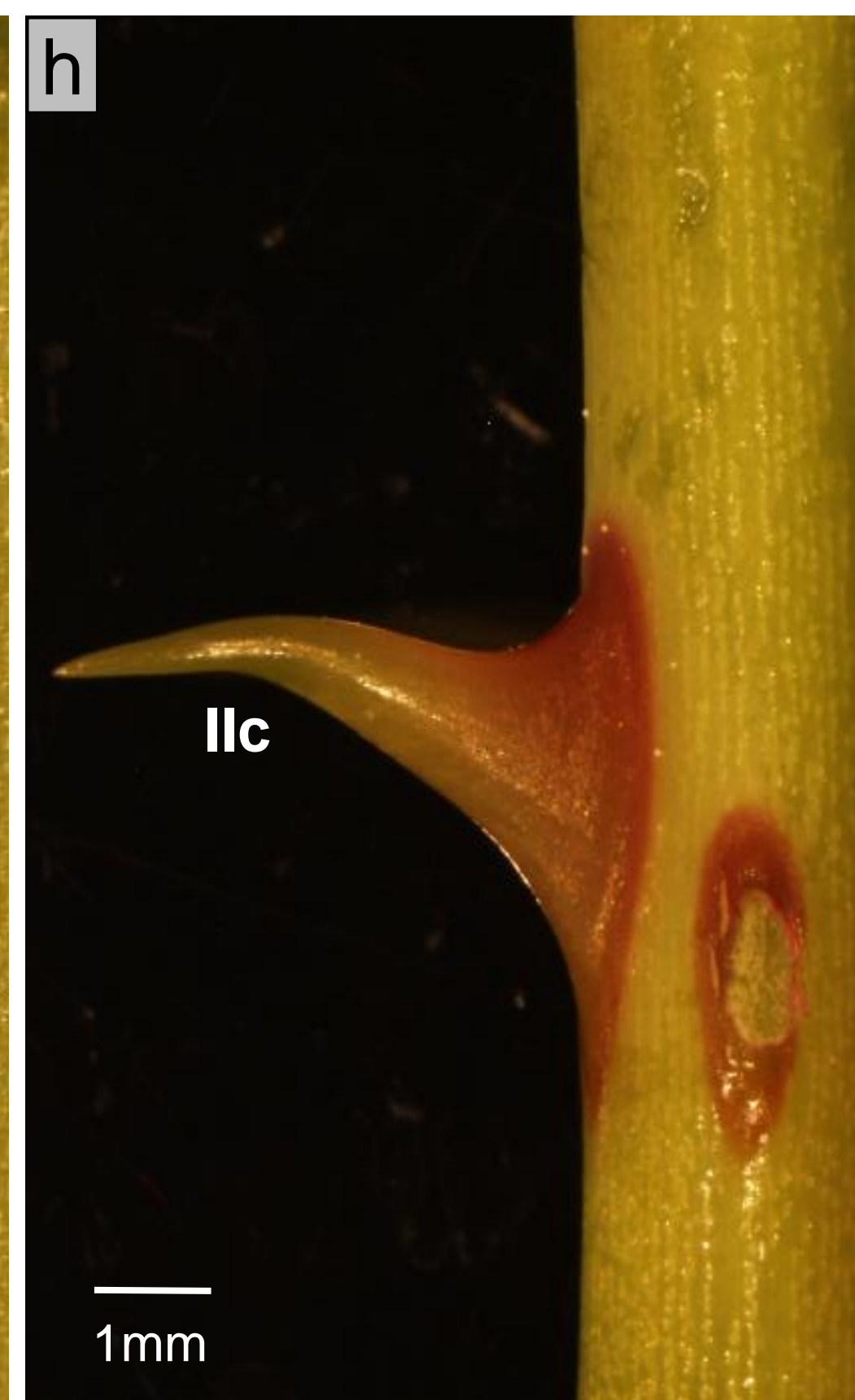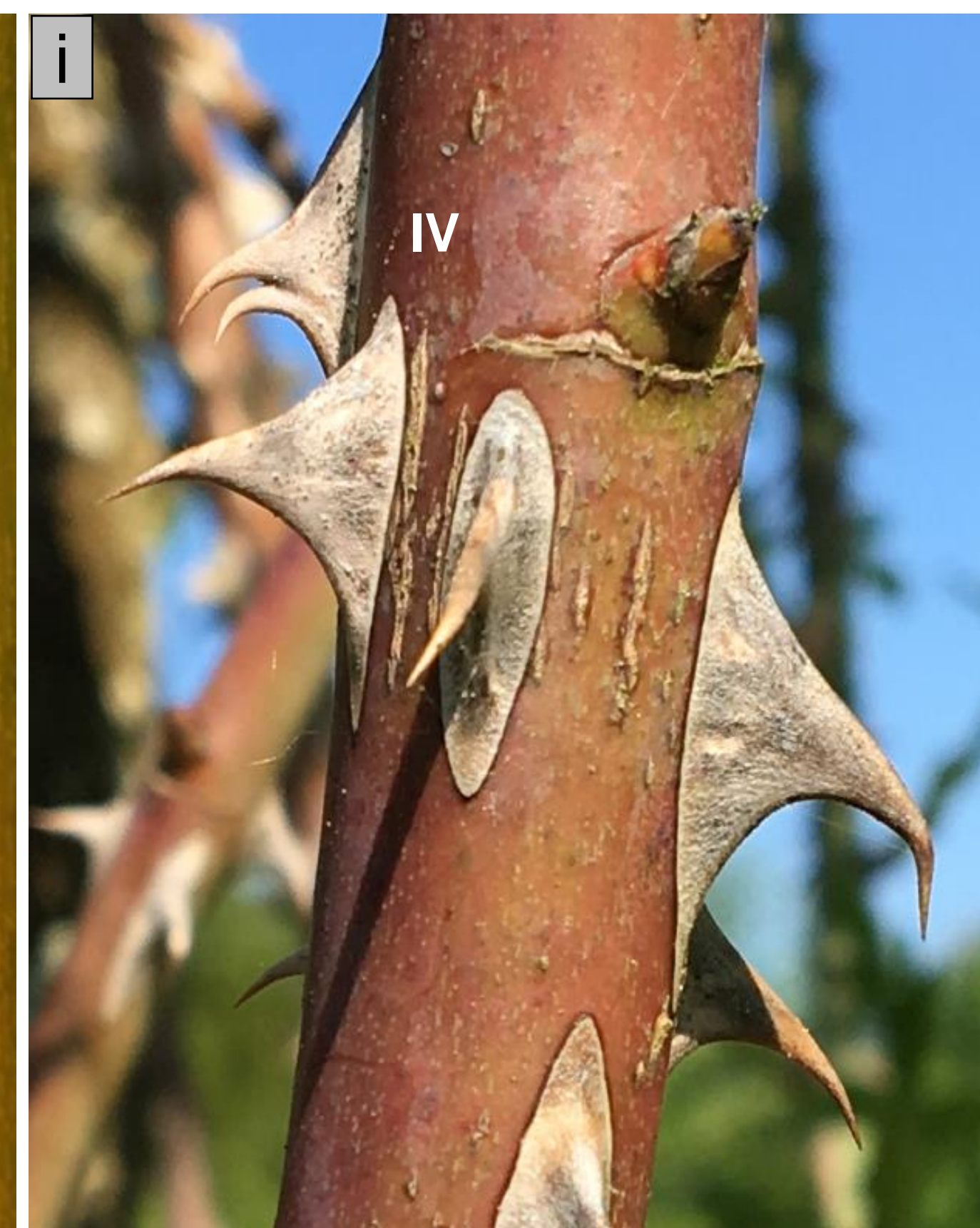

Supplement: Supplementary file 3 — Non-glandular prickle developmental process in R. ecae (a-e) and R. laxa (f-i) [file 41438_2021_689_MOESM3_ESM.pdf]

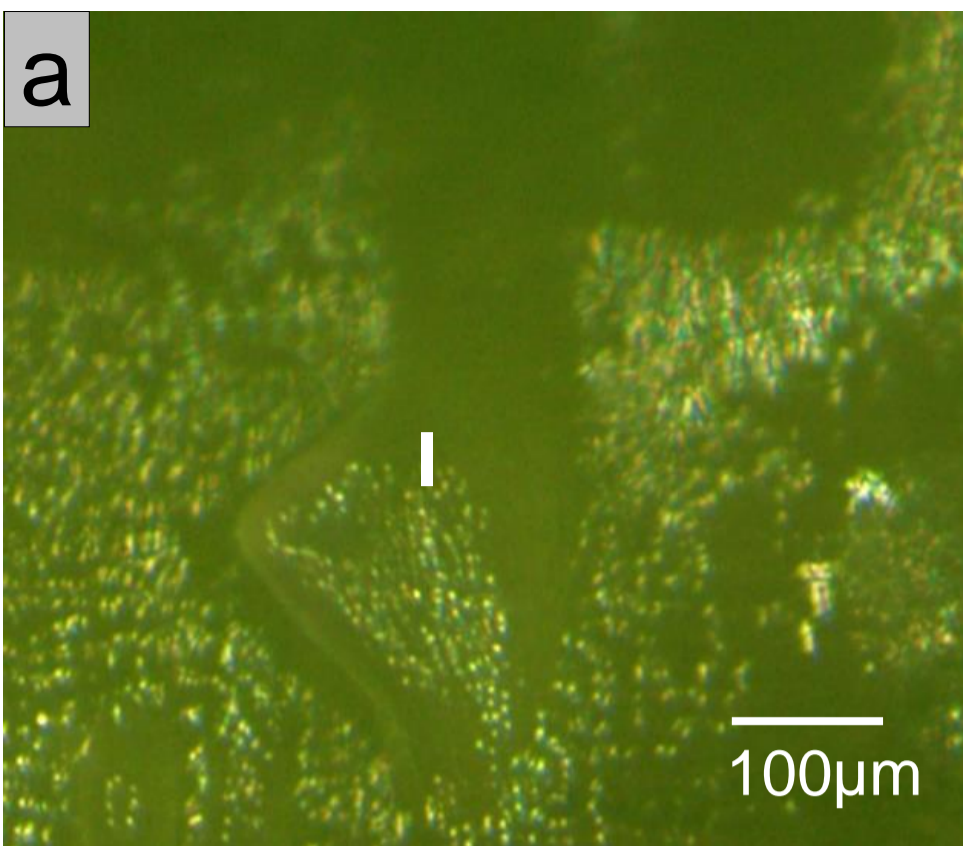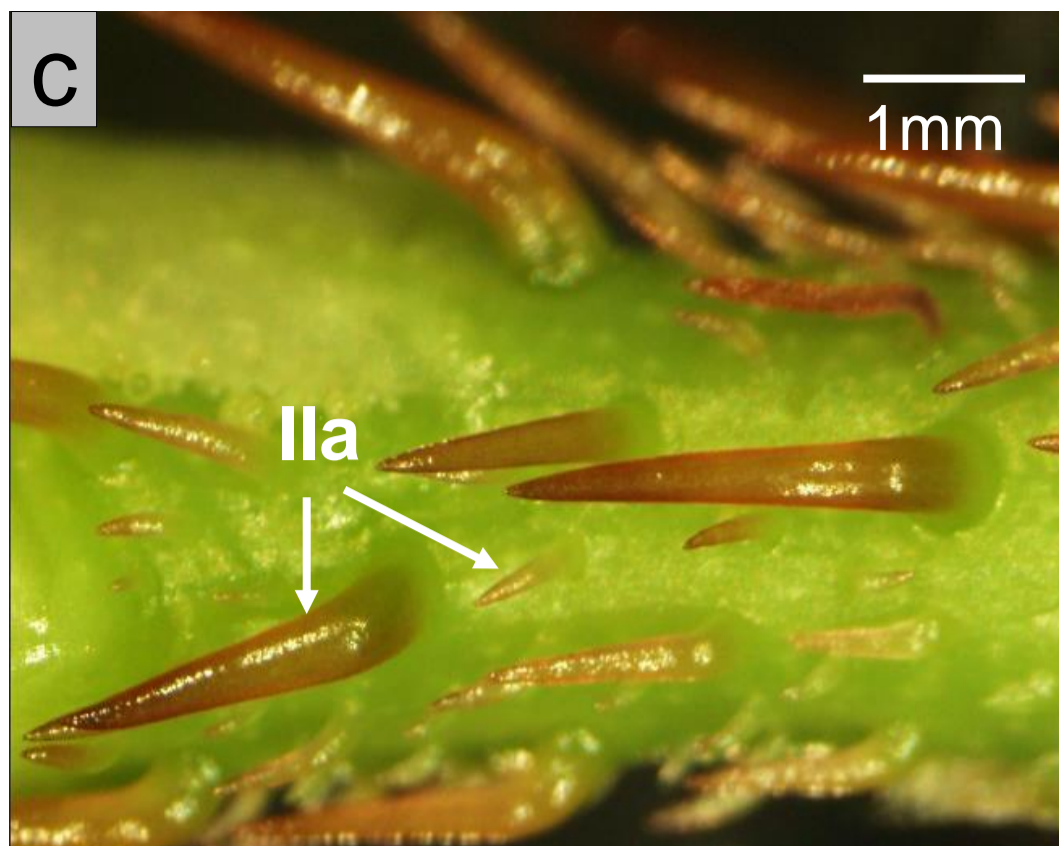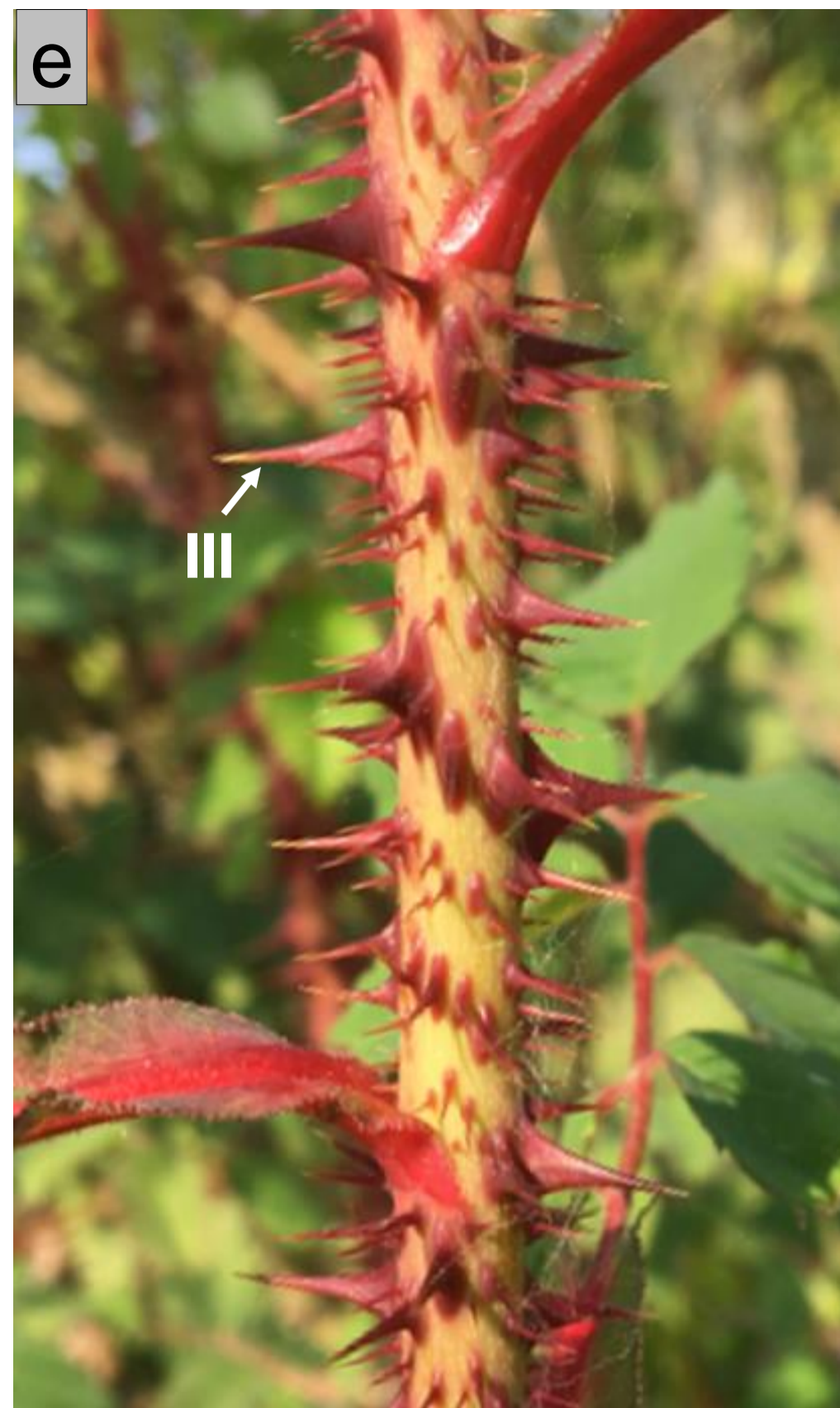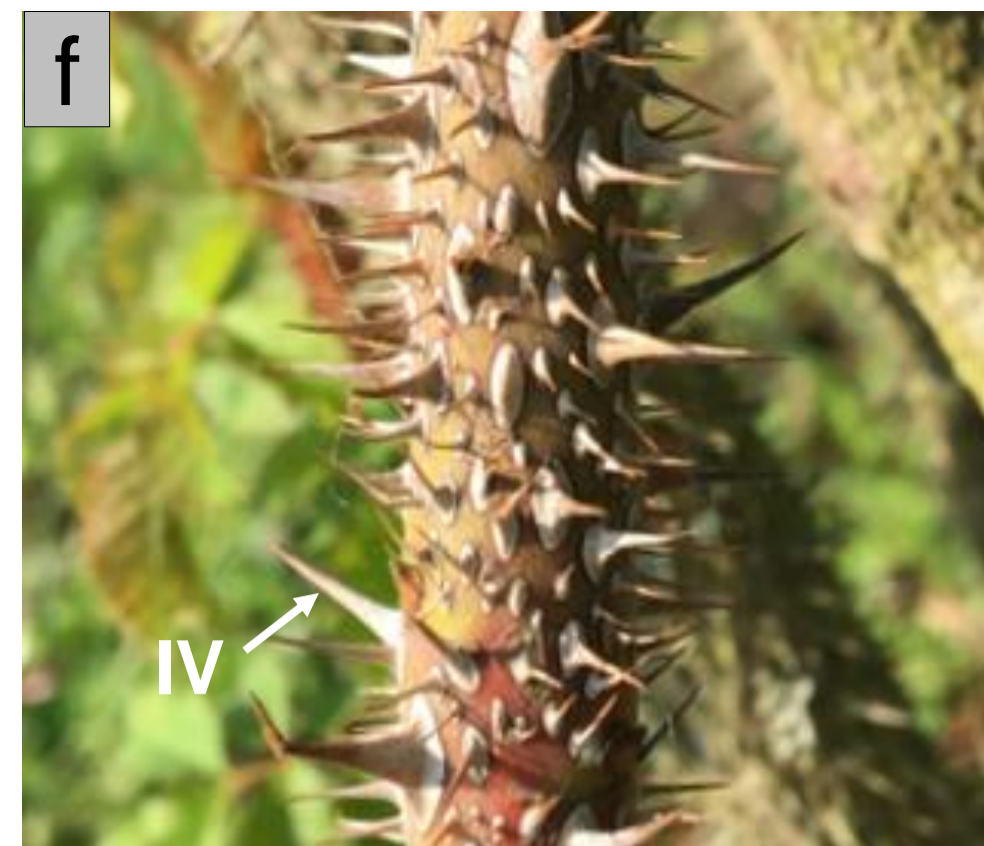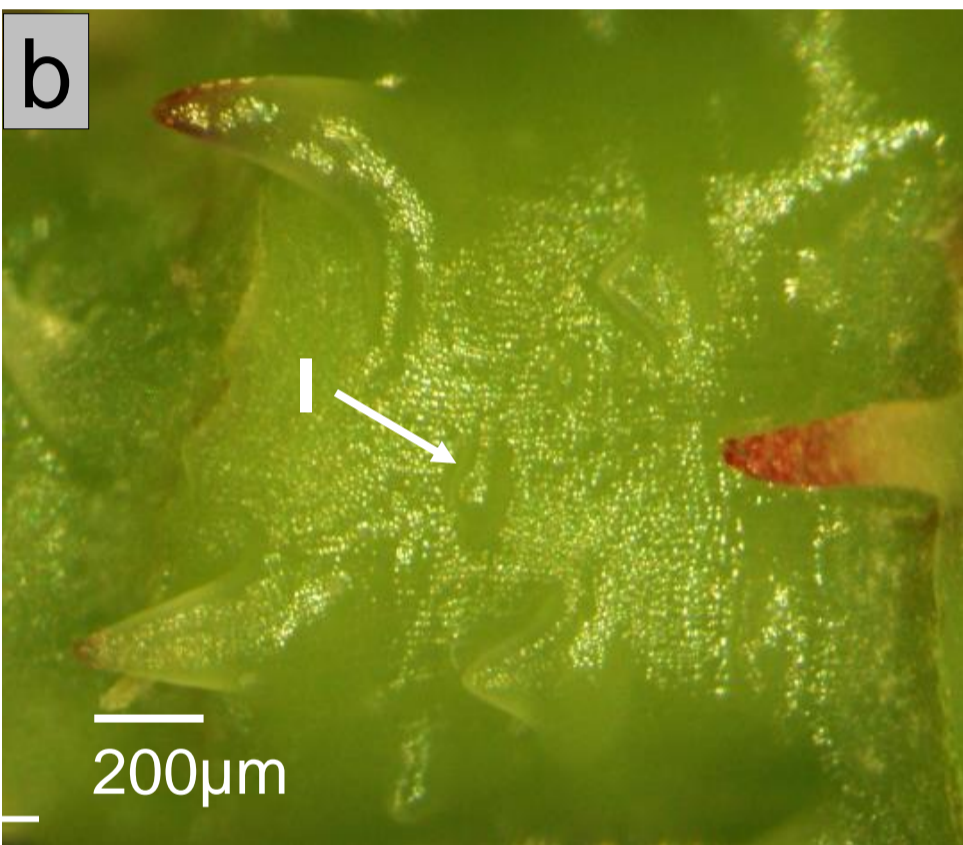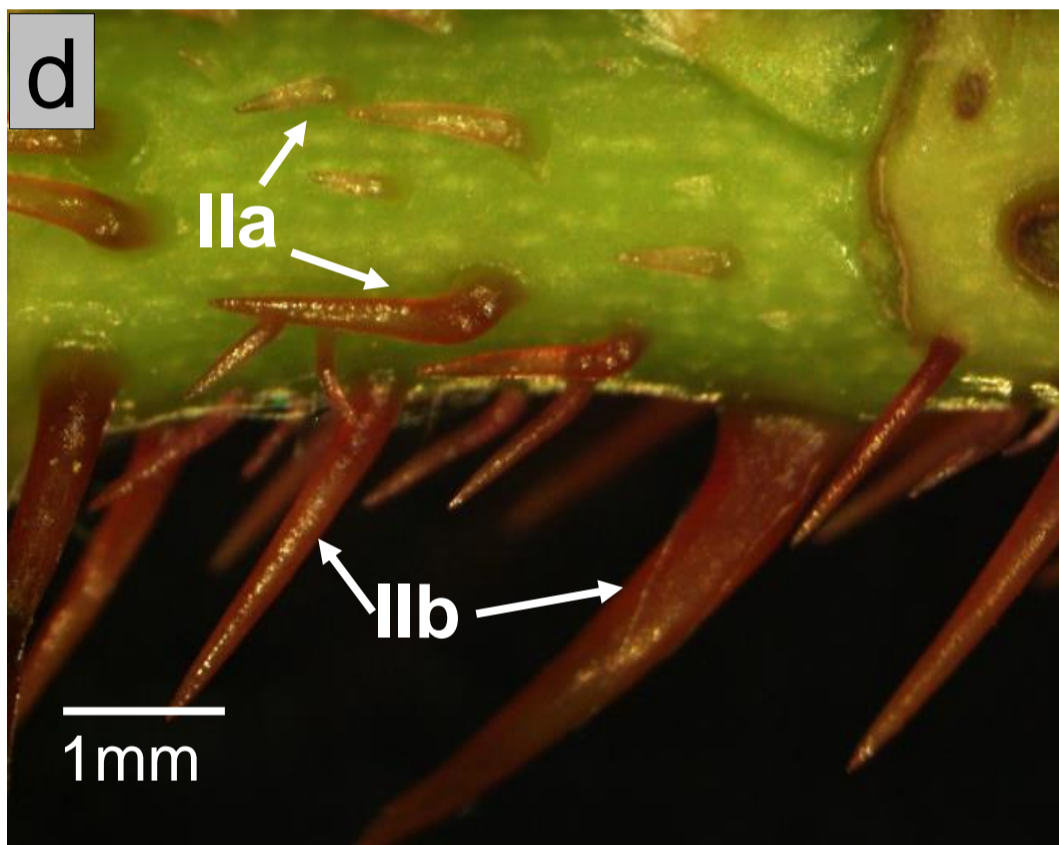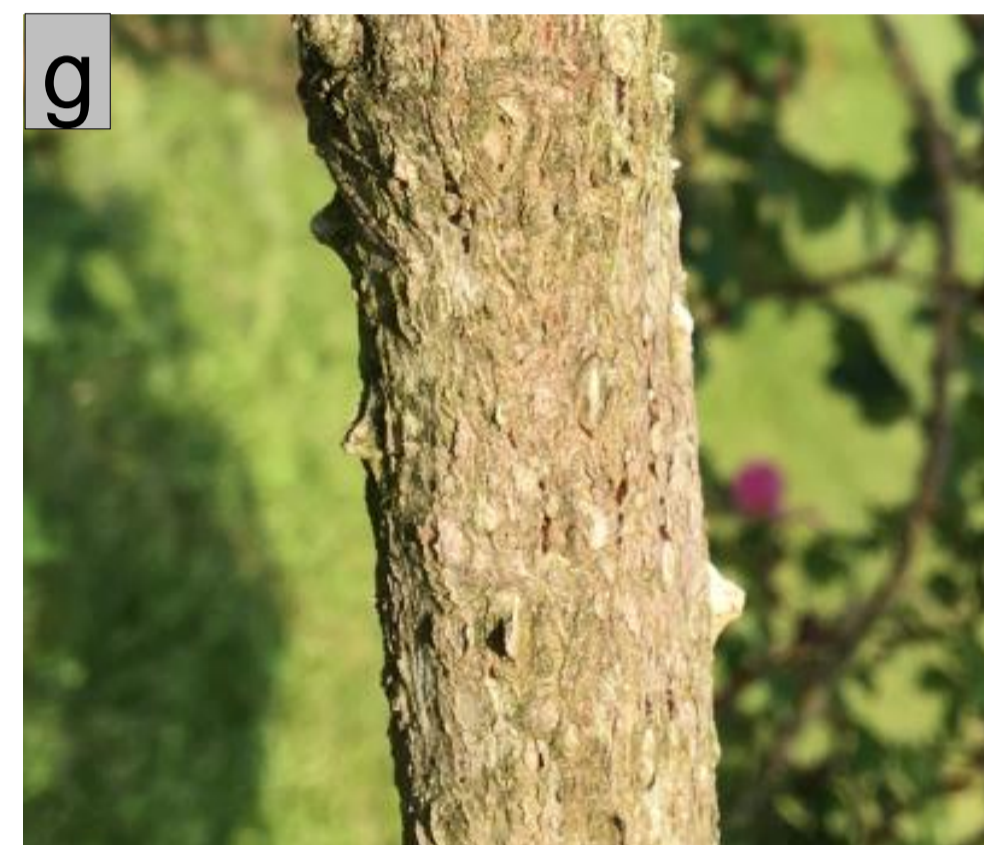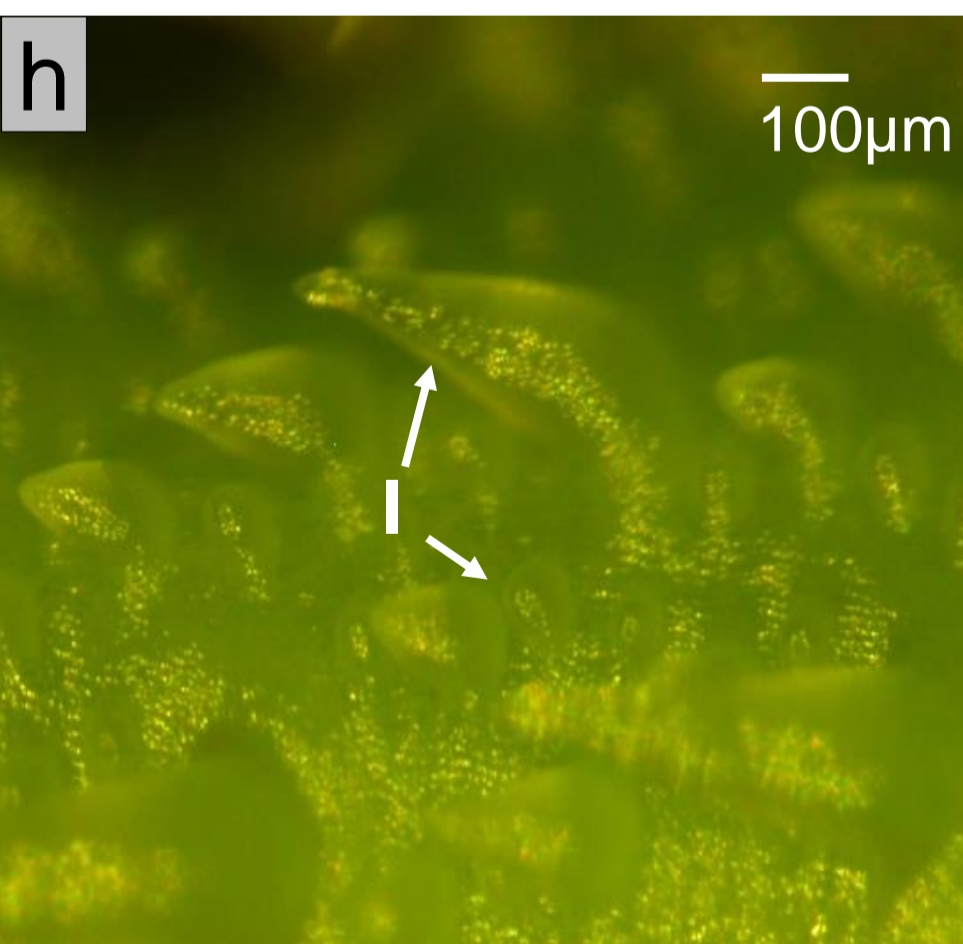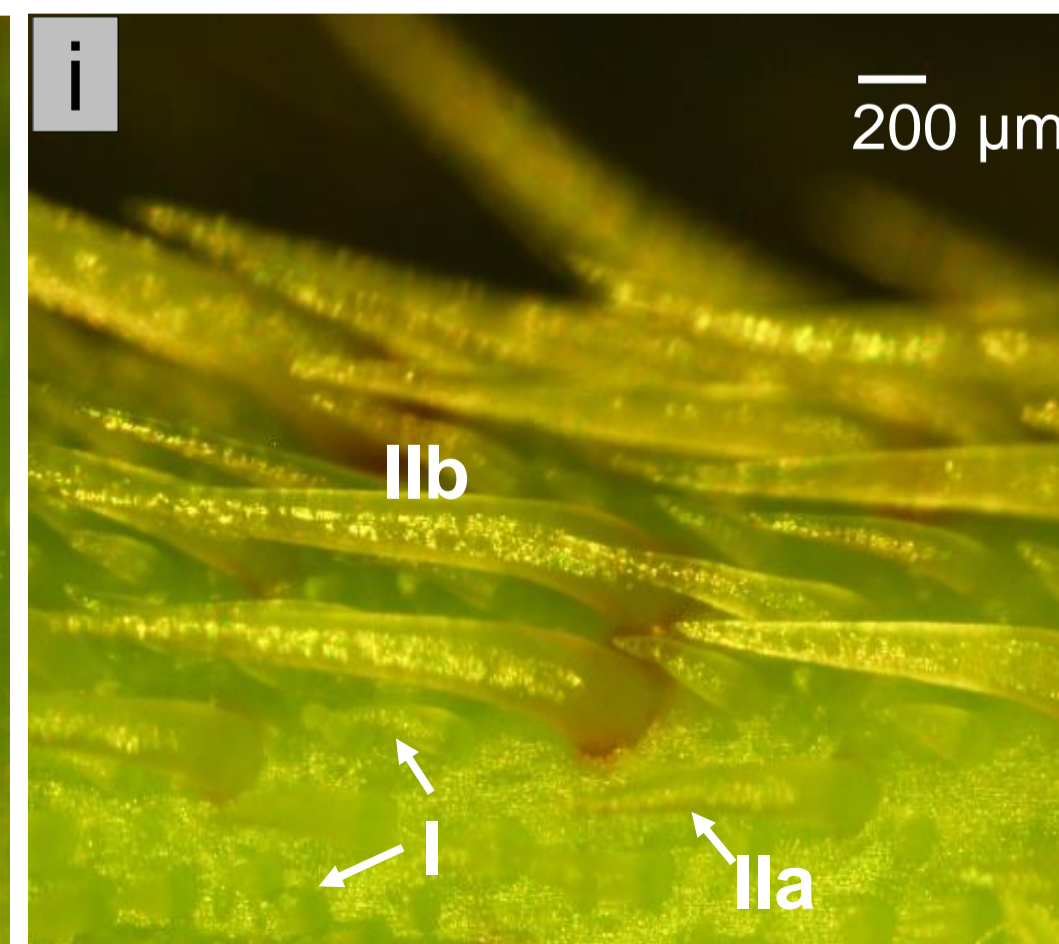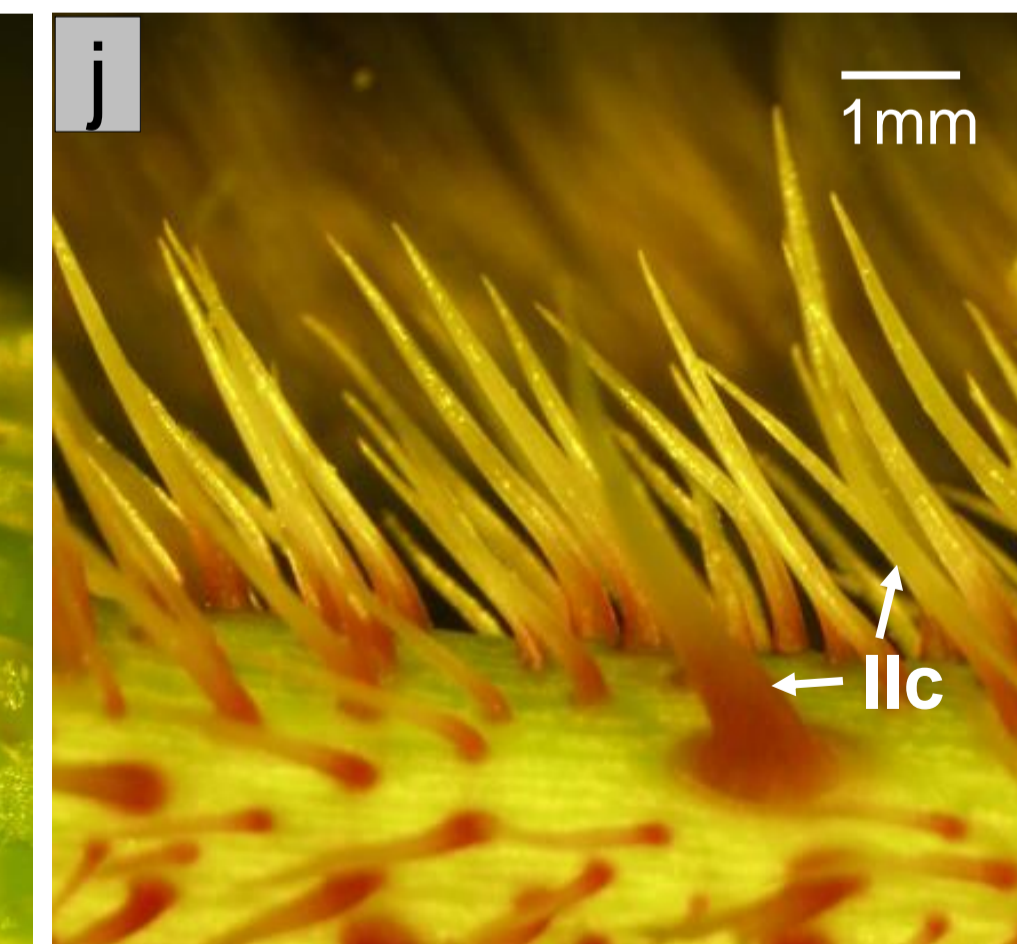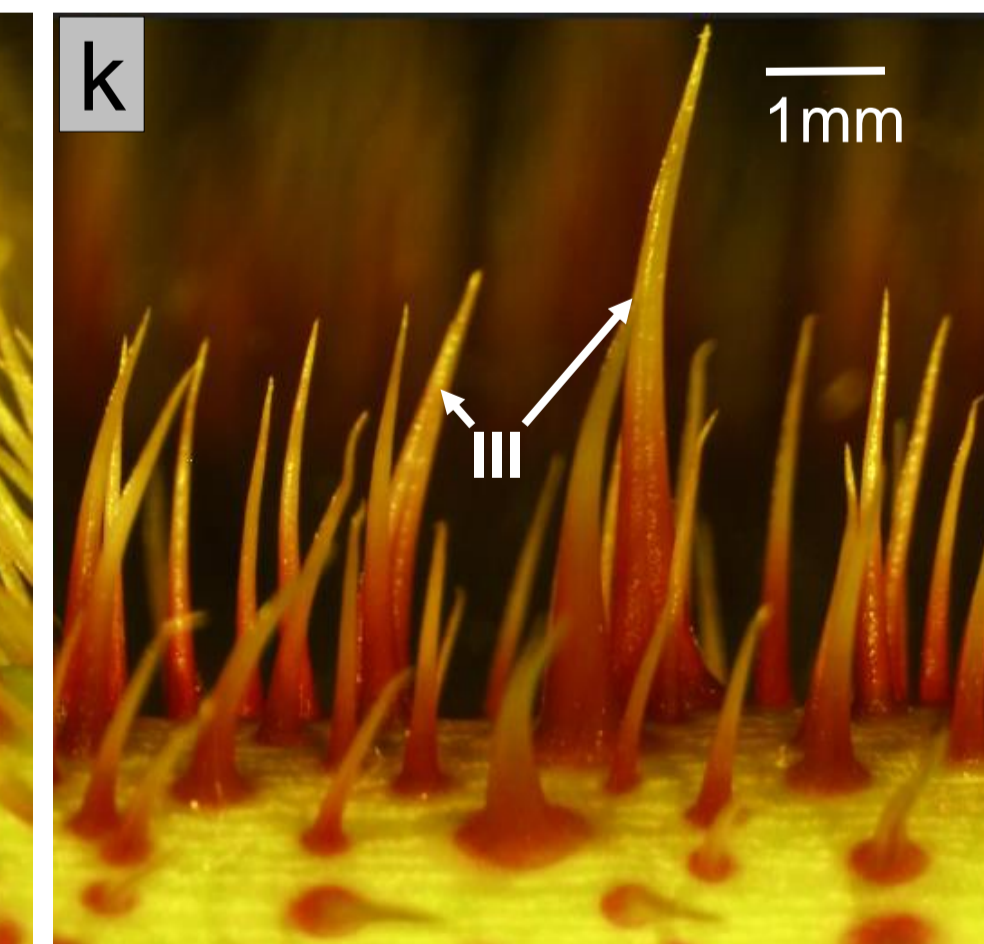

Supplement: Supplementary file 4 — Non-glandular prickles developmental process in R. sherardi (a-g) and R. moschata synstylae (h-k) [file 41438_2021_689_MOESM4_ESM.pdf]

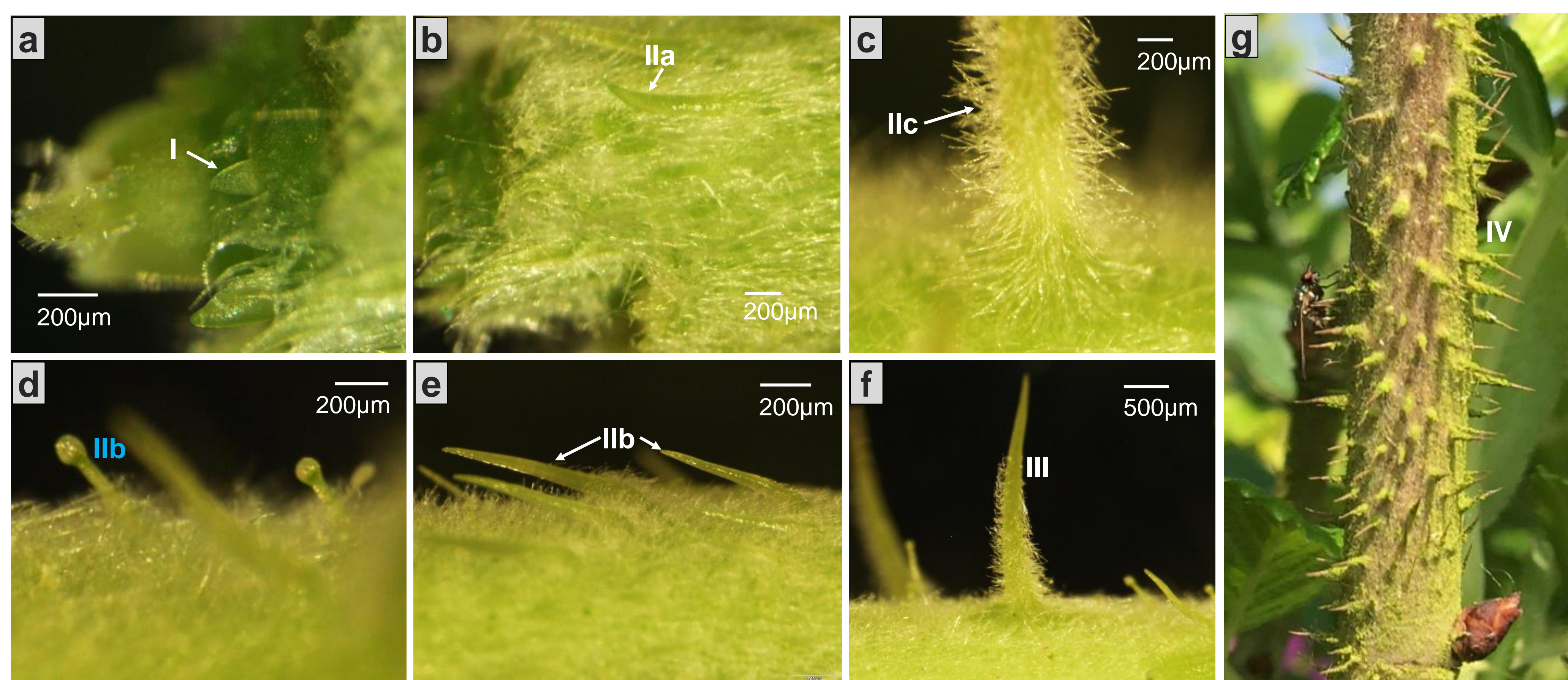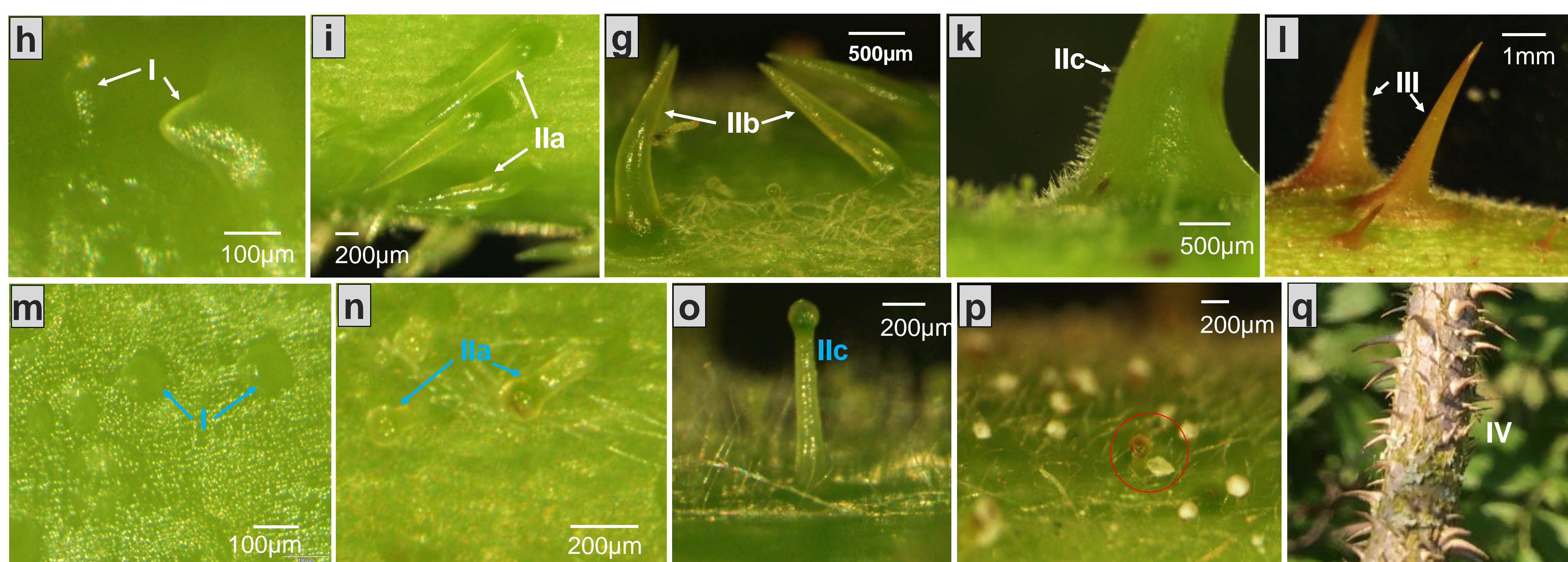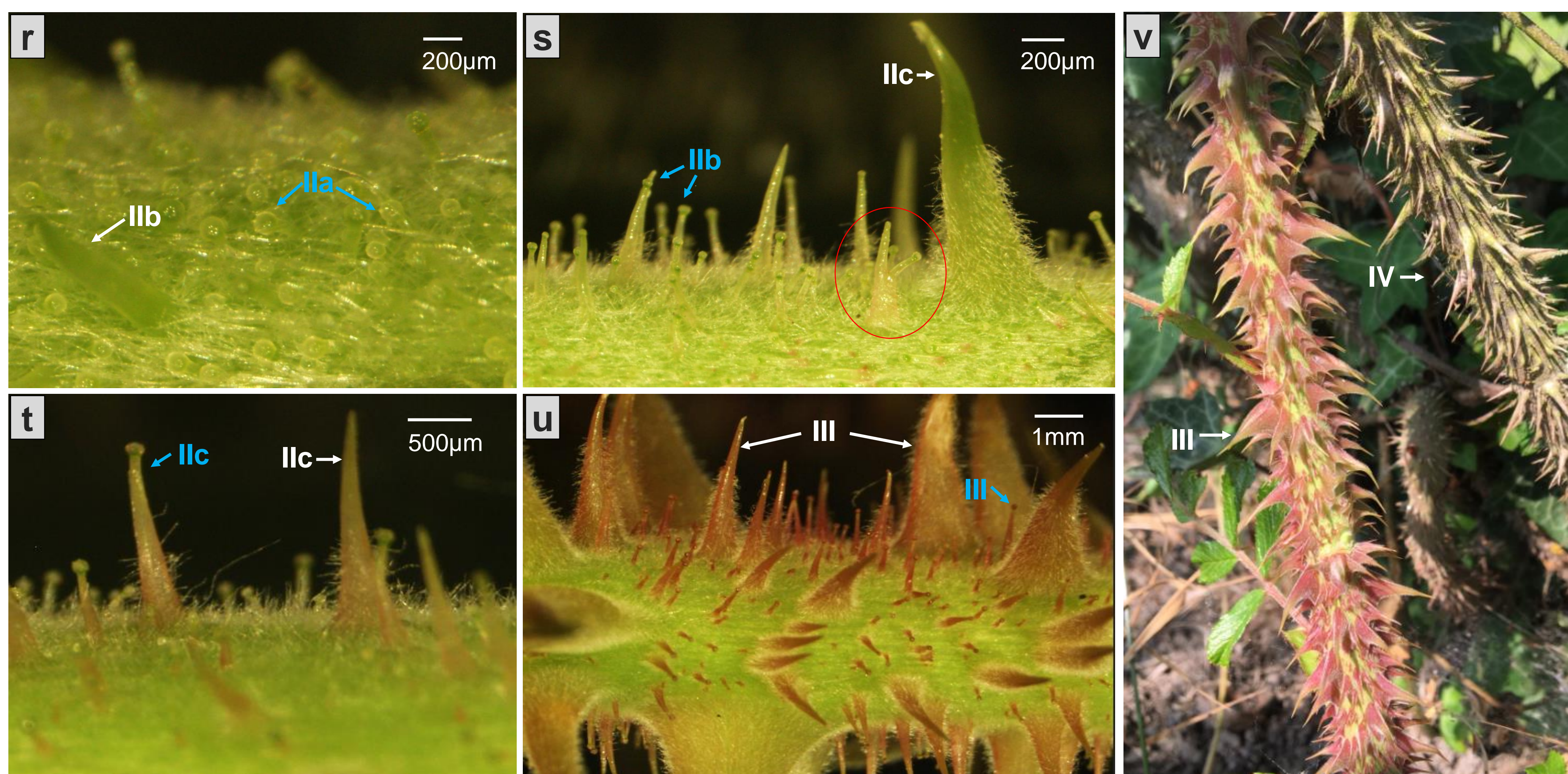

Supplement: Supplementary file 5 — Non-glandular and glandular prickles development in R. rugosa scabrosa (a-g), R. iwara (h-q) and R. ‘Grootendorst Supreme’(r-v) [file 41438_2021_689_MOESM5_ESM.pdf]

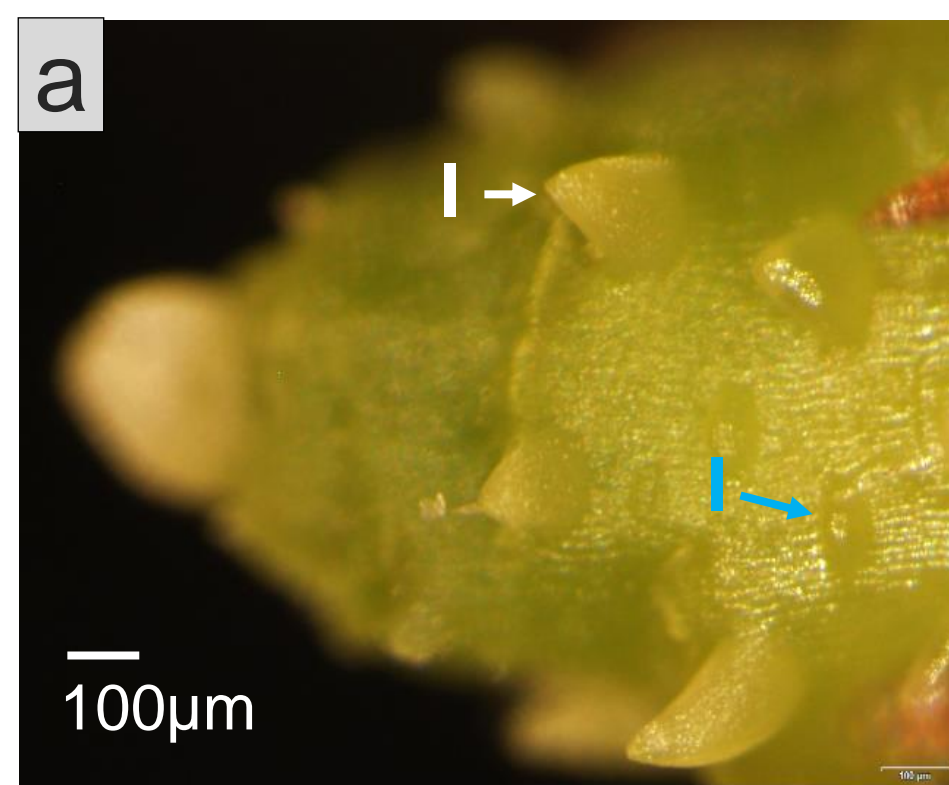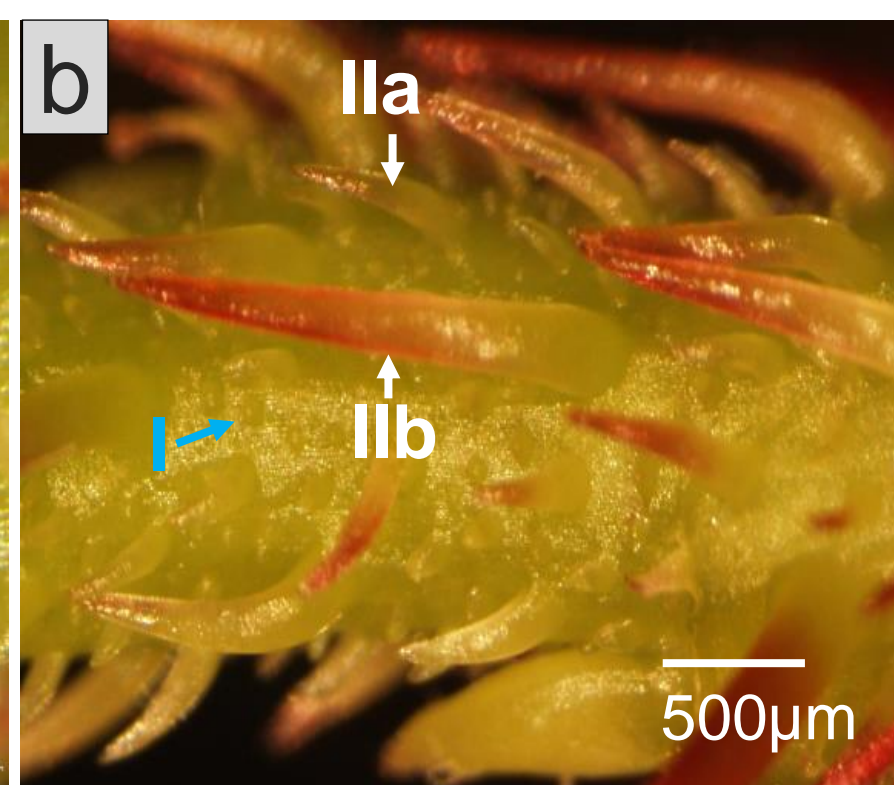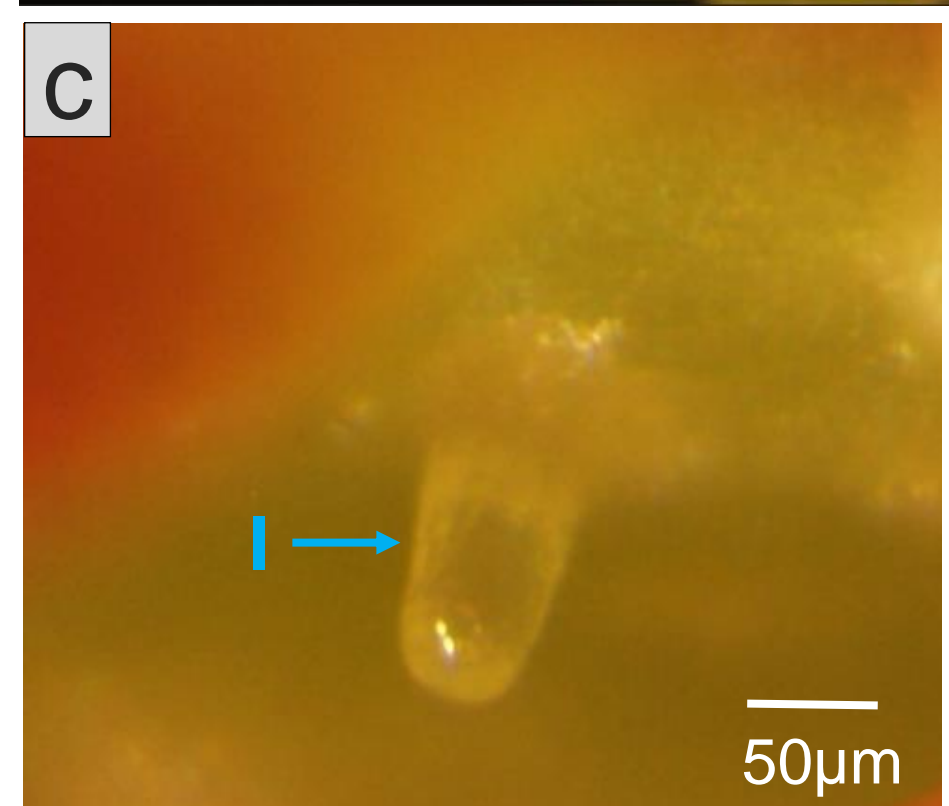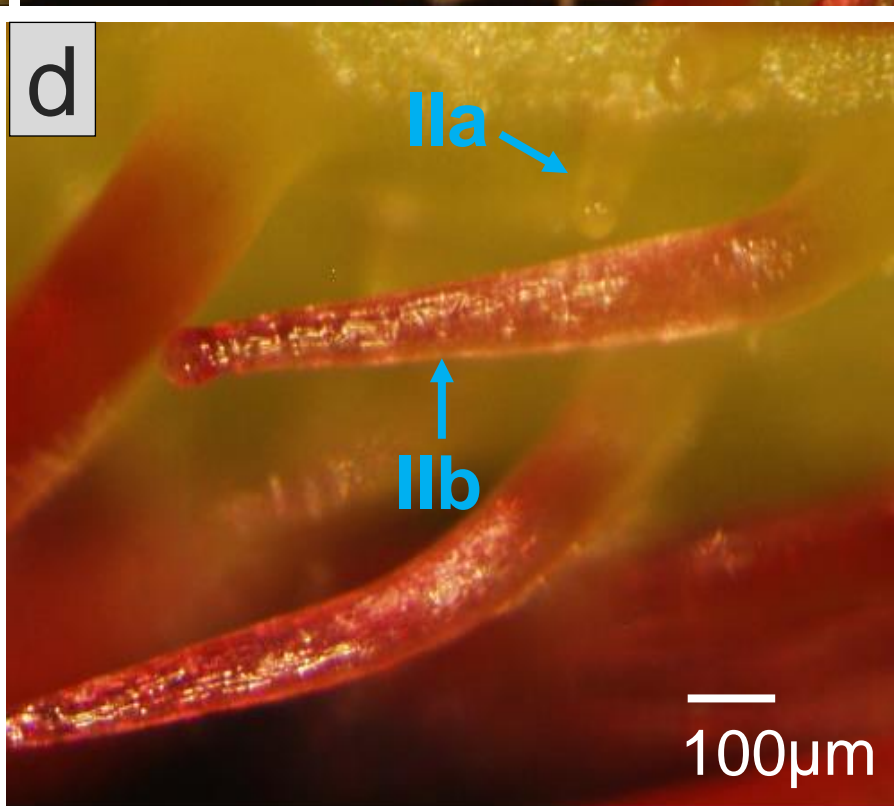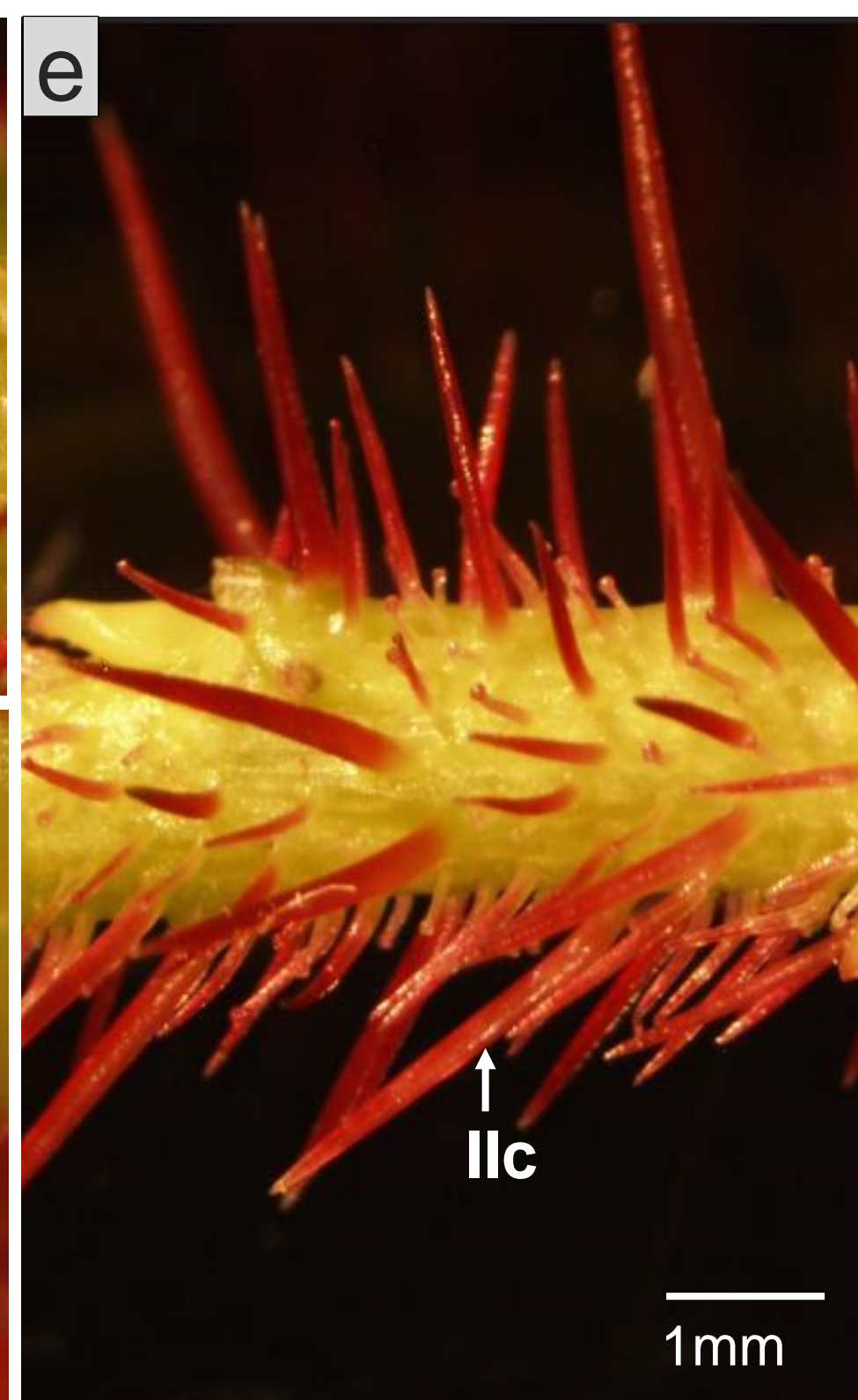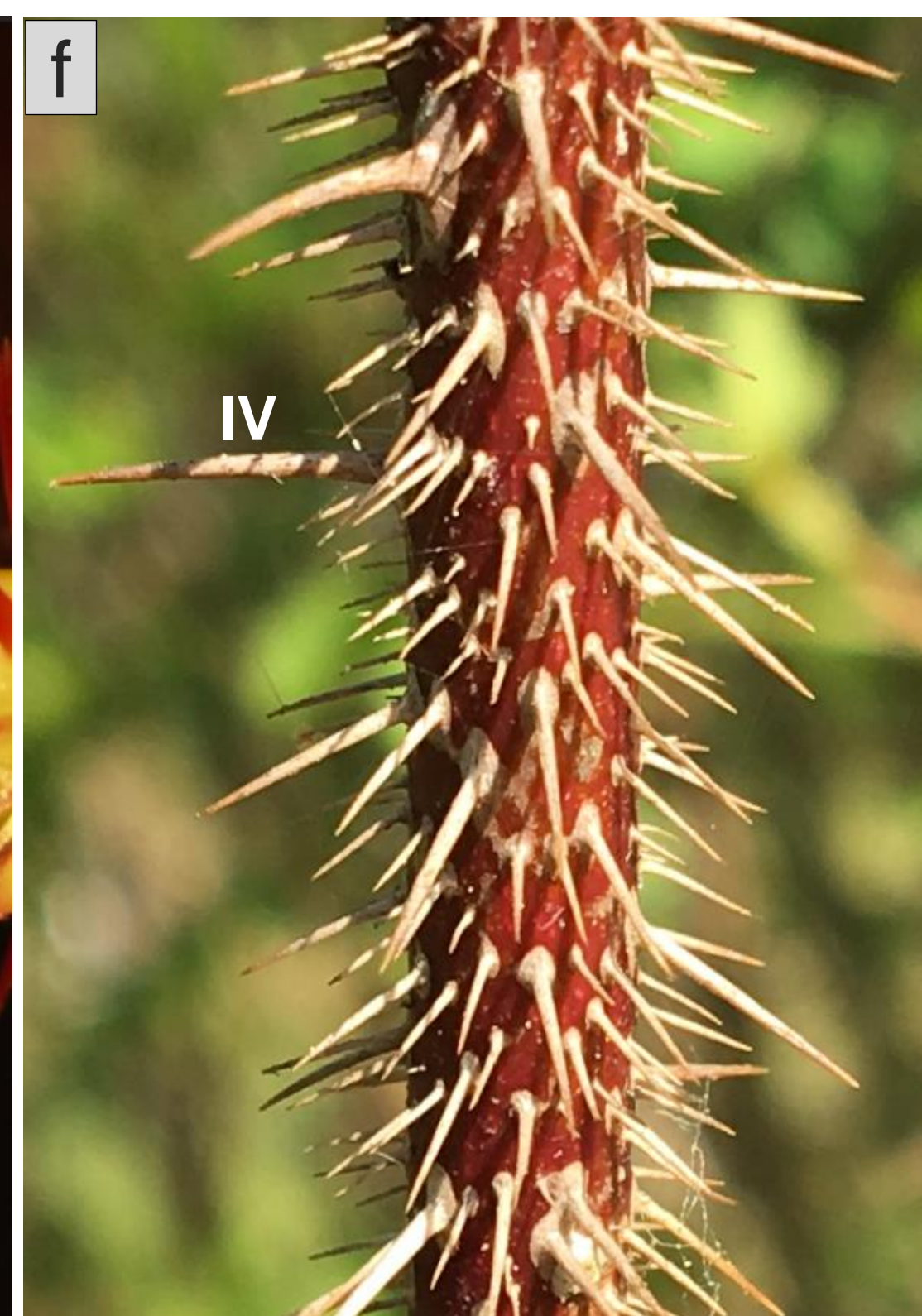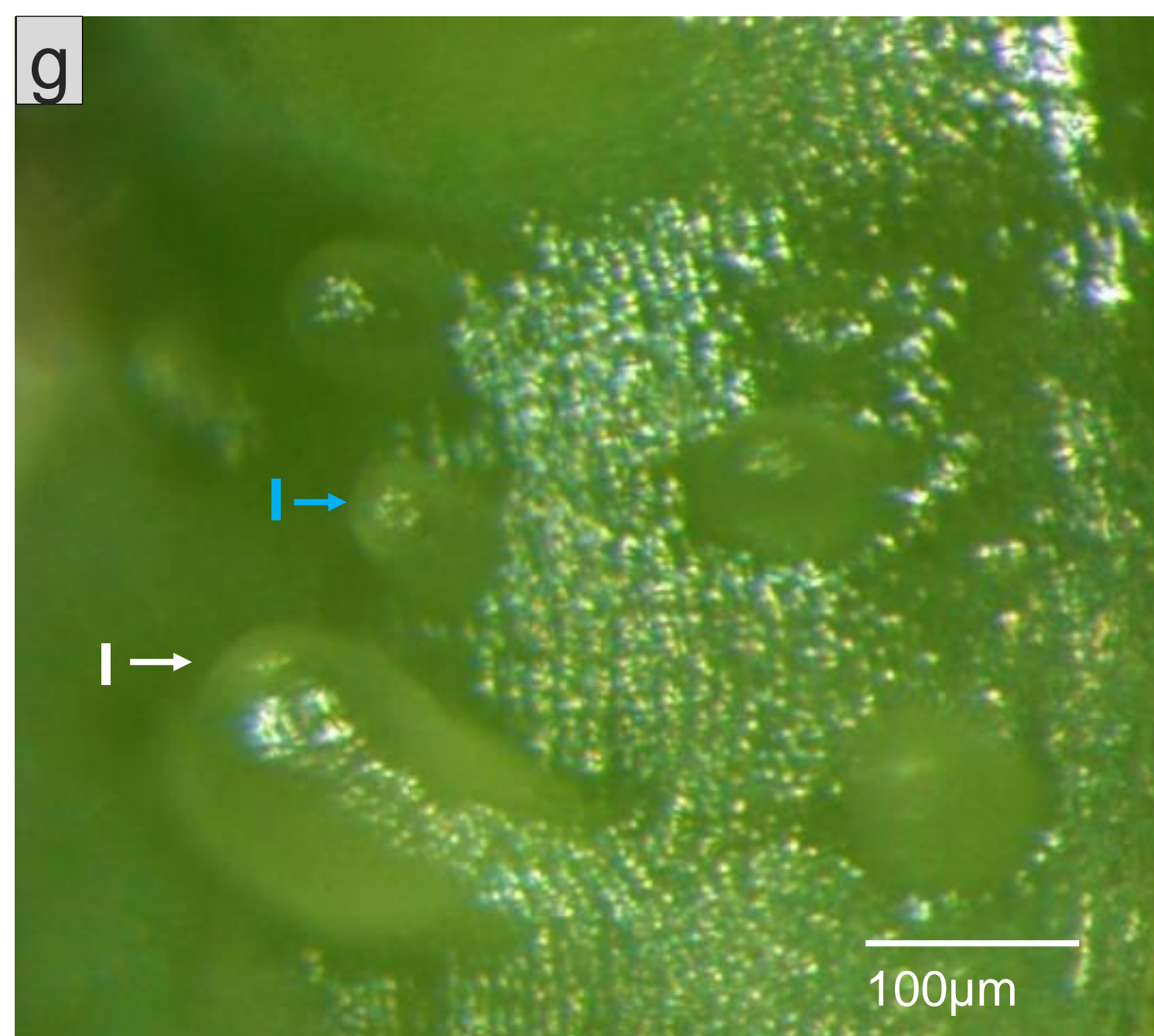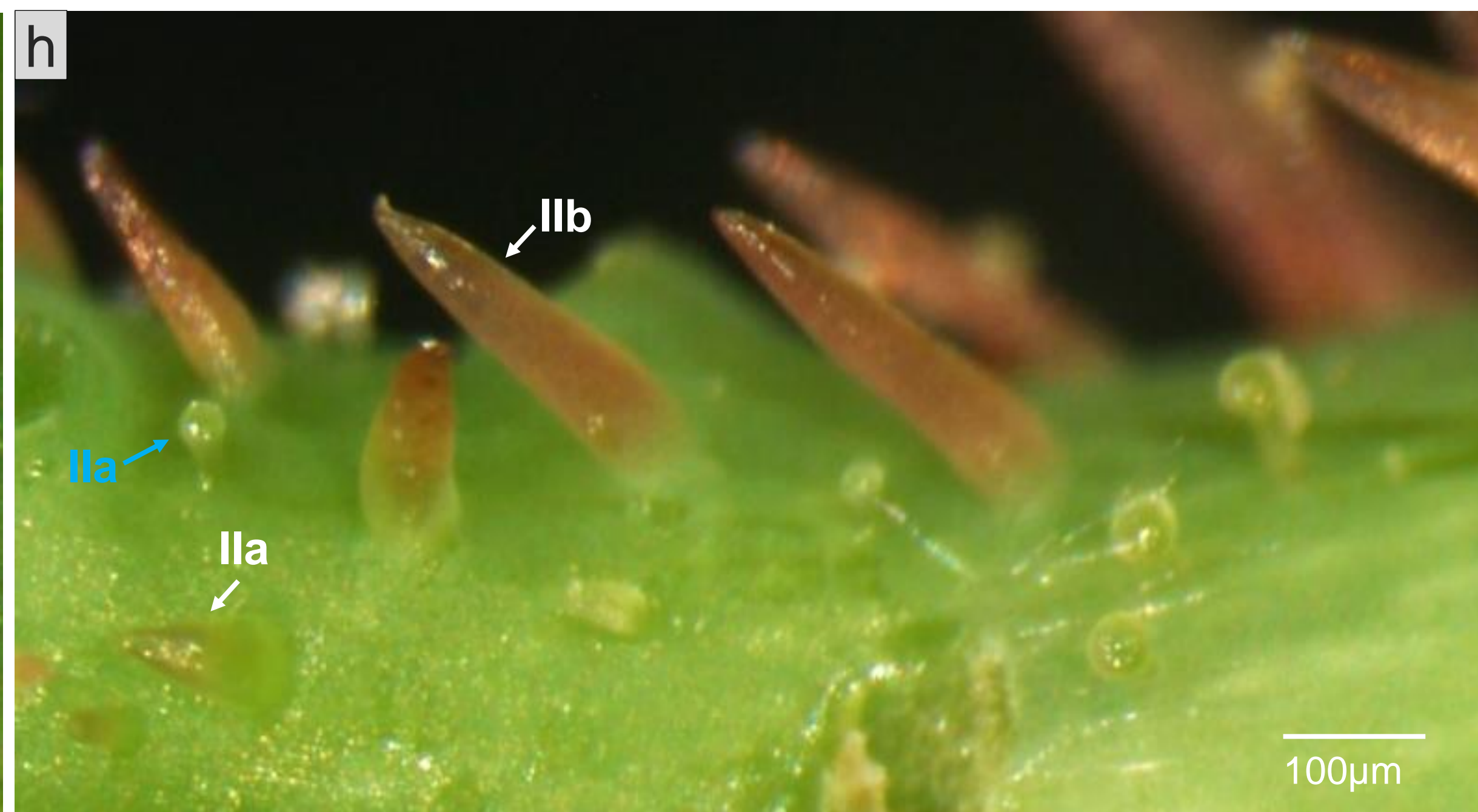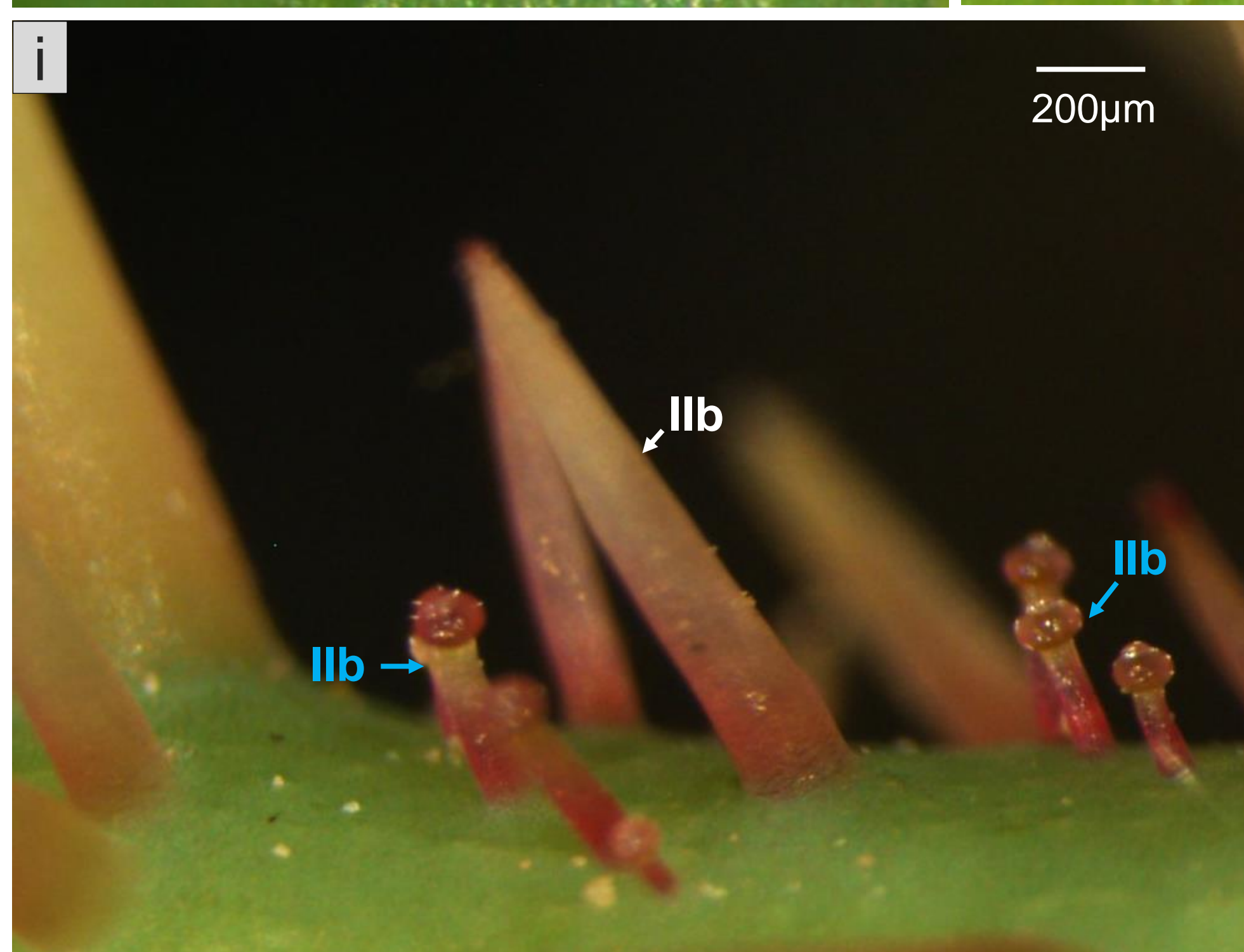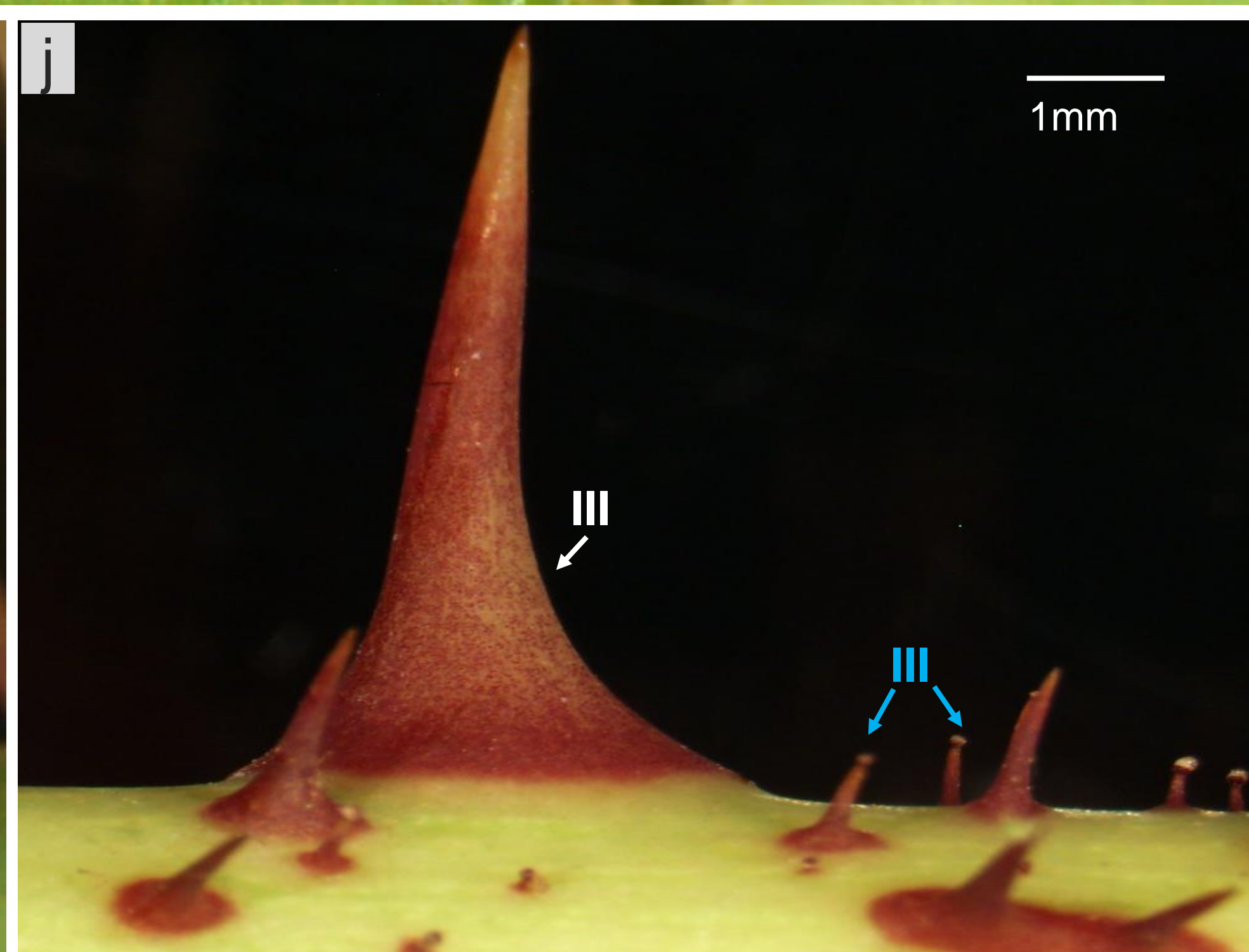

Supplement: Supplementary file 6 — Non-glandular and glandular prickle developmental process in R. rubella (a-f) and R. damascena (g-j) [file 41438_2021_689_MOESM6_ESM.pdf]
